# Bifurcations and multistability in inducible three-gene toggle switch networks

**Published:** 2025-09-29

**Authors:** Rebecca J. Rousseau, Rob Phillips

**Affiliations:** 1 Department of Physics, California Institute of Technology, Pasadena, CA 91125; 2 Division of Biology and Biological Engineering, California Institute of Technology, Pasadena, CA 91125

## Abstract

Control of transcription presides over a vast array of biological processes including through gene regulatory circuits that exhibit multistability. Two- and three-gene network motifs are often found to be critical parts of the repertoire of metabolic and developmental pathways. Theoretical models of these circuits, however, typically vary parameters such as dissociation constants, transcription rates, and degradation rates without specifying precisely how these parameters are controlled biologically. In this paper, we examine the role of effector molecules, which can alter the concentrations of the active transcription factors that control regulation, and are ubiquitous to regulatory processes across biological settings. We specifically consider allosteric regulation in the context of extending the standard bistable switch to three-gene networks, and explore the rich multistable dynamics exhibited in these architectures as a function of effector concentrations. We then study how the conditions required for tristability and more complex dynamics, and the bifurcations in dynamic phase space upon tuning effector concentrations, evolve under various interpretations of regulatory circuit mechanics, the underlying activity of inducers, and perturbations thereof. Notably, the biological mechanism by which we model effector control over dual-function proteins transforms not only the phenotypic trend of dynamic tuning but also the set of available dynamic regimes. In this way, we determine key parameters and regulatory features that drive phenotypic decisions, and offer an experimentally tunable structure for encoding inducible multistable behavior arising from both single and dual-function allosteric transcription factors.

## I. INTRODUCTION

Biological processes rely on intricate networks of gene interactions that together encode a multitude of possible cellular functions. Often, these genes produce proteins, called transcription factors, that alter the expression of other genes. Whether considering resource consumption in *E. coli* [1] or stages in animal development such as digit formation [2–4], vulval development [5, 6], or stem cell differentiation [7–9], such biological systems can precisely tune the relevant gene interactions toward many possible distinct phenotypes. This inherent multistability is crucial to cell function, allowing signaling from the environment, from metabolic resource constraints, or from other sources to induce systems toward what at times can be dramatically different end states.

Determining from experimental data alone the exact regulatory interactions that collectively drive multistable dynamics, however, is another matter. Indeed, the sensitivities of dynamics and steady state outcomes to both internal and external tuning are challenging to disentangle. To better understand these dynamics, it is useful to explore simpler motifs that establish the building blocks for multistability in higher-order networks.

One regulatory motif that has emerged as an integral component of natural and synthetic genetic circuit design is the bistable toggle switch, in which each of the two genes produces a protein that represses expression of the other. Study of the lysis vs. lysogeny decision in bacteriophage lambda in the 1960–80s established the significance of this motif experimentally [10–13], and subsequent research has modeled such motifs extensively using Hill models [14–16]. As a two-state model, however, the bistable switch sheds only partial light on the multistability of realistic phenotypic expression in cells.

Insights into the emergence of multistable dynamics in larger networks rely not only on the choice of model system but also on the approach to modeling these motifs. Existing literature on multistable cell fates during development, for example, has made significant strides by considering gene networks in terms of the Waddington landscape [17], and discusses the dynamic thresholds, or bifurcations, that can emerge from gene circuits described as attractor systems [18–21]. When linking this interpretation to mathematical models, however, such studies often represent systems using either a set of network-free flow equations or a potential gradient. These approaches are undoubtedly useful for exploring dynamic stability through the tuning of theoretically accessible internal parameters such as dissociation constants, production rates, and degradation rates. In this paper, we directly link these properties to experimentally accessible tuning knobs, and precisely specify how the relevant parameters are quantitatively controlled in living cells [22].

In fact, the activity of transcription factors often depends on the presence or absence of effector molecules [22–31]. When targeting a repressor, for example, an effector could bind and stabilize a protein in its inactive configuration, which suppresses its function and induces expression. Certain transcription factors are also known to have context-dependent behavior, acting as repressors at one promoter site along the DNA and activators at another [32, 33]. Exploring different interpretations of the mechanistic role of effectors may then reveal critical differences in tuned expression that allow for complementary experimental investigation.

We thus focus on examining the role of the effector concentration as a dynamical system parameter. Tuning inducer concentration alone, we can then directly observe corresponding shifts in dynamics. This framework offers a perspective for parametrization that is accessible to the experimentalist and also inherently to cell activity itself. For example, researchers can directly input external non-metabolizable effectors to study the dynamic shifts observed in theory [34], and cells can also naturally use one or more effectors, with two inducers driving cell fate determination in the lac operon and in cell differentiation during development, among other settings [16, 35]. Given the extensive experimental evidence emphasizing the significance of effectors as natural tuning knobs, we aim to theoretically examine how dynamics respond to explicit definitions for effectors and for the biological mechanism of induction.

**FIG. 1:**
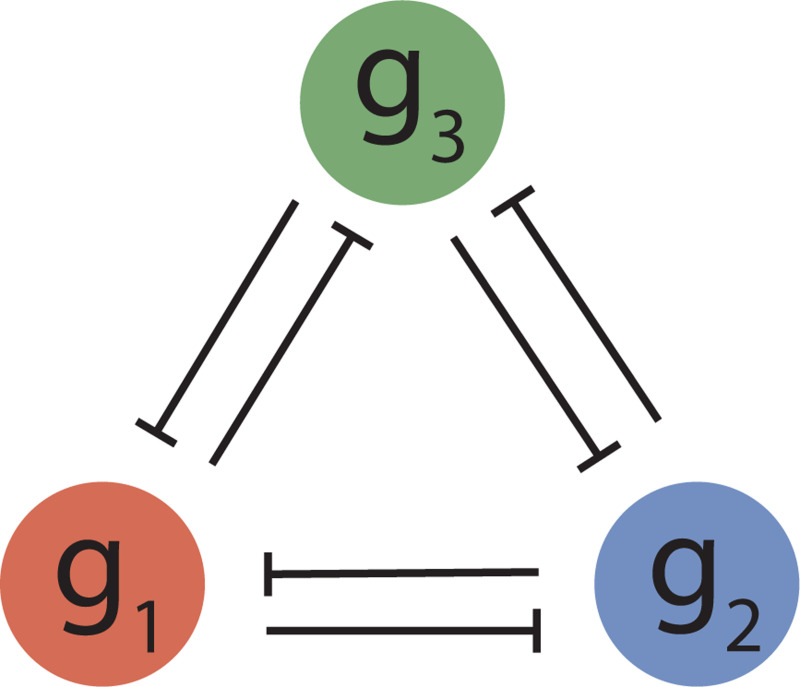
Network of three mutually-repressing genes. Transcribing each gene $g_{i}$ produces a repressor with average concentration denoted by $R_{i}$.

To analyze the roles of effector molecules in the emergence of multistability, we specifically study extensions of the toggle switch to three genes as shown in Figure 1. These motifs remain simple enough to visualize and physically interpret, yet also provide an additional layer of tunable gene interaction parameters that can allow more complex multistable dynamics – in this case, tristability and beyond. In exploring how a set of more than two genes couple to collectively decide among multiple possible cell fates, we gain insights into how these interactions drive dynamics in larger and more complex networks.

The three-gene toggle switch provides a powerful basis for examining the emergence of tristability and higher-order multistability in a range of biological contexts. The motif is most directly useful in a synthetic setting, where dynamic shifts can be tuned precisely through experiments. However, the model can also help uncover how various sets of three (or more) key genes interact to generate multistable cell fates in nature. For example, experimental work has observed sets of three “master regulator” genes that interact to drive protein expression toward three or more distinct cell fate phenotypes, such as in T helper cell differentiation [36] and stem cell differentiation [37].

Recent work has already begun to examine the comprehensive picture of stable expression in such three-gene toggles [38, 39]. Our study considers various approaches to induction in an alternative Hill model for the three-gene toggle. Within this reframed context for tuning dynamics, we then investigate not only how the mechanisms of induction shift transcription factor activities and stable state expressions, but also how the dynamic topologies of the resulting fixed point landscapes evolve.

We begin in Section II by defining our model for the three-gene toggle switch, and how to mathematically incorporate the regulatory activity of transcription factors as allosterically-inducible proteins. From this initial framework, Section III then demonstrates the different types of dynamics possible as inducer concentrations rise. In Section IV we highlight how the thresholds, i.e., bifurcations, that separate different dynamic regimes shift when perturbed away from an initial assumed symmetry in gene interactions. Section V explores the impacts of additional induction and self-activation on the complexity of dynamics and the number of stable steady states possible for the three-gene toggle switch. In the latter case in particular, we find that the biological interpretation of the mechanism for dual repression and self-activation, whether involving binding exclusivity or the effector’s role, can significantly affect the types of dynamics that unfold. In so doing, our work sheds light on the complexity of dynamics possible from simple changes in modeling choices, and on just how precisely inducer concentrations must be tuned under different conditions for a system to access various dynamic regimes. The diverse range of input-output responses demonstrated across the different three-gene networks presented here only further underscores the importance of understanding how these results generalize to higher-order gene regulatory architectures.

## II. THE THREE-GENE TOGGLE SWITCH

We first consider the most direct extension of the two-gene bistable toggle switch to three genes, as depicted in Fig. 1. Transcribing a given gene $g_{i}$ generates a protein $R_{i}$ called a transcription factor, which can in turn bind to the promoter region of either remaining gene. In this particular incarnation of the model, the transcription factor prevents RNA polymerase (RNAP) from binding and initiating transcription, thereby repressing expression. We begin by discussing how to model the dynamics of this system of repressors before demonstrating the role of induction in governing how dynamical behavior evolves as a function of inducer concentration(s).

### A. The baseline model

The modeling of stability in basic switches and other regulatory motifs has long been explored using the tools of dynamical systems [15, 40–43]. To describe the expression patterns that arise from the three-gene toggle switch architecture shown in Fig. 1, we define a dynamic equation for each transcription factor repressor $R_{i}$ of the form

(1) $$\frac{dR_{i}}{dt}=a_{i}p_{i}^{\mathrm{expr}}\left(R_{i},\left\{R_{j\neq i}\right\}\right)-\frac{1}{\tau_{i}}R_{i}.$$

This general model form assumes that the dynamics of gene transcription, i.e., the production of mRNA transcripts, evolves at a timescale such that production can be measured equivalently from the concentration of output repressor $R_{i}$. The first term of Eqn. 1 characterizes protein production at a maximal rate $a_{i}$, and depends on the probability $p_{i}^{\mathrm{expr}}$ that expression can occur. The second term accounts for protein degradation at a rate $1/\tau_{i}$.

**FIG. 2:**
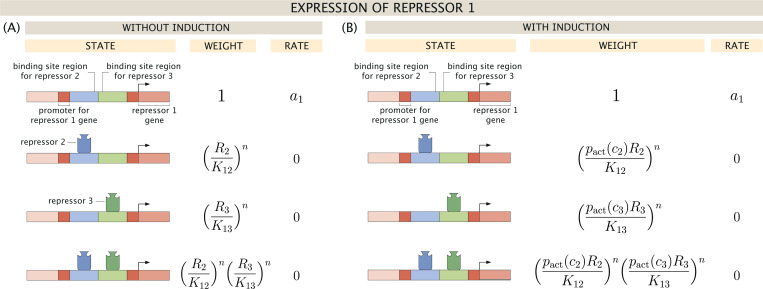
Expression of repressor 1 in the three-gene toggle switch. Equivalent definitions apply to expression of repressors 2 and 3. (A) Thermodynamic states, weights, and rates for expression of repressor 1. The regulating repressors ($R_{2}$ and $R_{3}$) bind non-exclusively to the target promoter region to suppress gene transcription. Each repressor $R_{i}$ binds at the promoter region for repressor $R_{1}$ with affinity $K_{1i}$. (B) Thermodynamic states, weights, and rates for expression of repressor 1 in the presence of inducers. Expression now depends on the active concentration of each repressor $R_{i}$, which is determined by a distinct inducer at concentration $c_{i}$, defining the probability of activity $p_{\text{act}}\left(c_{i}\right)$.

Fig. 2(A) breaks down the possible regulatory states and corresponding statistical weights that contribute to the probability of expression $p_{i}^{\mathrm{expr}}$. This approach to describing biological regulation draws from well-established statistical mechanical principles [44–54]. From this table, and equivalent definitions for expression of repressors 2 and 3, we define the dynamics of this three-gene circuit by the set of differential equations

(2) $$\frac{dR_{1}}{dt}=\frac{a_{1}}{\left[1+{\left(\frac{R_{2}}{K_{12}}\right)}^{n}\right]\left[1+{\left(\frac{R_{3}}{K_{13}}\right)}^{n}\right]}-\frac{1}{\tau_{1}}R_{1},$$

(3) $$\frac{dR_{2}}{dt}=\frac{a_{2}}{\left[1+{\left(\frac{R_{1}}{K_{21}}\right)}^{n}\right]\left[1+{\left(\frac{R_{3}}{K_{23}}\right)}^{n}\right]}-\frac{1}{\tau_{2}}R_{2},$$

(4) $$\frac{dR_{3}}{dt}=\frac{a_{3}}{\left[1+{\left(\frac{R_{1}}{K_{31}}\right)}^{n}\right]\left[1+{\left(\frac{R_{2}}{K_{32}}\right)}^{n}\right]}-\frac{1}{\tau_{3}}R_{3}.$$

Each repressor $R_{j}$ binds non-exclusively with affinity $K_{ij}$ to the promoter region for the gene that expresses repressor $R_{i}$. This repression coefficient $K_{ij}$ thus reflects the characteristic concentration of repressor $R_{j}$ required to strongly regulate $R_{i}$’s expression. In other words, when $R_{j}=K_{ij}$, the produced concentration of repressor $R_{i}$ is half its maximum possible value.

Note that these equations describe transcription factor binding phenomenologically through a Hill function model with coefficient n [55, 56]. This approach coarse-grains out the molecular detail of precise binding site occupancies found in thermodynamic models, instead representing the high cooperativity-limit of the thermodynamic model. The Hill coefficient n thus does not necessarily correspond to the number of bound repressor molecules, but rather measures the sensitivity of output response to binding.

While there are advantages to the specificity of thermodynamic models [57], using them to evaluate stability profiles quickly becomes intractable in higher-dimensional networks, including those analyzed here. We therefore take a Hill model approach for the remainder of the discussion. Even without full knowledge of precise binding site occupancies, the Hill function offers a strong fit to empirical data for gene regulatory dynamics [14, 58]. The model also reflects the Hopfield barrier for the sharpness of input-output response [59], meaning that for a given coefficient n, the Hill model reflects the strongest, most sigmoidal input-output response possible for a system without energy expenditure. The Hill approach thus allows us to investigate dynamics under the most input-sensitive model conditions, and to observe its maximum capability for dynamic complexity.

We now have a model for the toggle switch that depends on various theoretically accessible parameters, specifically dissociation constants $K_{ij}$, production rates $a_{i}$, and degradation rates $1/\tau_{i}$. As pointed out previously, however, these parameters as written are not easily accessible experimentally, and are disconnected from the underlying biology that controls them within living cells. While the current model indicates that regulation is controlled by the total concentrations of transcription factors, these proteins are often themselves controlled by the allosteric binding of effector molecules. Cells therefore respond to changes in the number of *active* transcription factors.

In the following subsections we will use statistical mechanics to outline the role of effector molecules (specifically inducers) in defining transcription factor activity.

### B. A statistical mechanical model for allosteric induction

Shifting to an allosteric description of gene regulation, we now argue that gene expression depends not on the total concentration of the regulating transcription factor, $\left[{\text{TF}}_{\text{tot}}\right]$, but rather on the *active* concentration thereof, i.e.,

(5) $$\left[{\text{TF}}_{\text{act}}\right]=p_{\text{act}}(c)\left[{\text{TF}}_{\text{tot}}\right],$$

where $p_{\text{act}}(c)$ is the probability that the transcription factor is active as a function of effector concentration c. This probability can be defined by the Monod-WymanChangeux (MWC) model [22–26, 29–31], which states that when an effector at concentration c can bind allosterically at m sites on a transcription factor, the probability that the transcription factor is active is

(6) $$p_{\text{act}}(c)=\frac{{\left(1+\frac{c}{K_{A}}\right)}^{m}}{{\left(1+\frac{c}{K_{A}}\right)}^{m}+e^{-\beta \text{Δ}\epsilon }{\left(1+\frac{c}{K_{I}}\right)}^{m}},$$

where $K_{A}$ and $K_{I}$ are the dissociation constants for active and inactive repressor states, respectively, $\beta =1/k_{B}T$, and $\text{Δ}\epsilon =\epsilon_{I}-\epsilon_{A}$ is the energy difference between inactive $\left(\epsilon_{I}\right)$ and active $\left(\epsilon_{A}\right)$ states. Repressors are driven toward the inactive state upon effector binding when $K_{I}<K_{A}$. Effectors obeying this condition are referred to as inducers. Conversely, when $K_{I}>K_{A}$, repressors are driven toward the active state, and effectors act as corepressors. Examples of both phenomena have been experimentally isolated in nature [60–62], but for simplicity we will assume the case of $K_{I}<K_{A}$ in what follows and refer exclusively to inducers.

**FIG. 3:**
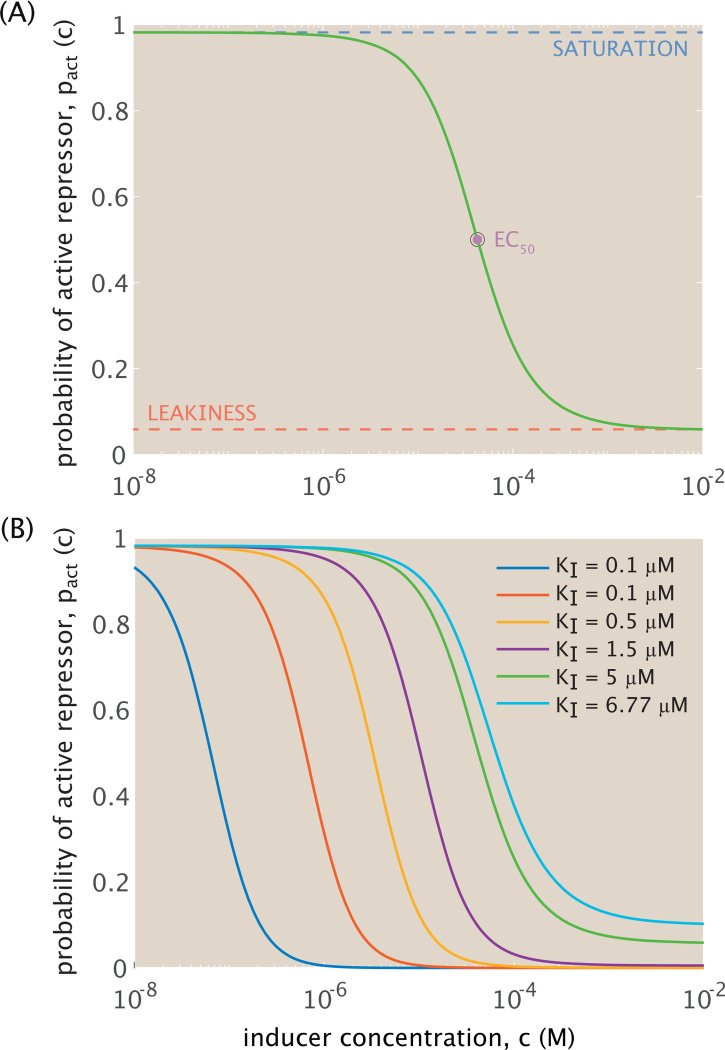
Activity of repressors as a function of inducer concentration c, for which $K_{I}<K_{A}$. (A) The probability of active repressor as a function of inducer concentration, with m=2, $\epsilon =4\;k_{B}T$, $K_{A}=150\;\mu M$, and $K_{I}=5\;\mu M$. The saturation and leakiness limits are denoted in blue and orange, respectively, and the $EC_{50}$, i.e., inducer concentration at which the probability is half maximal, is marked in purple. (B) Evolution of probability for different values of $K_{I}$, with the curve in panel (A) shown again in green. The shift in probability spans the range of allowable $K_{I}$ values for the specified parameter set up to the boundary at $K_{I}=6.77\;\mu M$, and assumes a fractional error of at most q=0.1 in saturation and leakiness.

Fig. 3(A) plots one possible probability curve $p_{\text{act}}(c)$ in the induction setting with $K_{I}<K_{A}$ for a fixed set of parameters. This function follows standard sigmoidal behavior, with a sufficient increase in inducer concentration rendering repressor inactive. Depending on how we define parameters, we can alter several key properties of the input-output response from this probability function. The leakiness represents the amount of activity at saturating concentrations of inducer, i.e., $p_{\text{act}}(c\to \infty )$, and the saturation conversely denotes the amount of activity in the absence of inducer $\left(p_{\text{act}}(c=0)\right)$. The inducer concentration at which the probability is half maximal is denoted as the $EC_{50}$ value, and the sharpness of the input-output response curve at this inflection point increases with an increasing Hill coefficient n. The MWC parameters chosen therefore determine how effectively inducers suppress the activity of repressors and the sensitivity of the output response to an increase in inducer concentration.

We determine the biologically permissible ranges for these MWC parameters $K_{I}$, $K_{A}$, and Δϵ) by making several assumptions. First, the absence of inducer does not imply the complete absence of inactive repressor, for there may be some natural fractional error q. We suppose, then, that in the absence of inducer,

(7) $$p_{\text{act}}(c=0)\geq 1-q,$$

or alternatively,

(8) $$1-p_{\text{act}}(c=0)=1-\frac{1}{1+e^{-\beta \text{Δ}\epsilon }}=\frac{1}{1+e^{\beta \text{Δ}\epsilon }}\leq q.$$

Rearranging Eqn. 8, we obtain a condition for the energy difference Δϵ between the inactive and active repressor configurations that

(9) $$e^{-\beta \text{Δ}\epsilon }\leq \frac{q}{1-q}.$$

It follows that for the probability of activity to perfectly saturate at unity in the absence of inducer, with q=0, the energy of the inactive repressor state must significantly exceed that of the active state so that Δϵ→∞ and the repressor is always active.

Gene expression is also generally “leaky,” meaning there is typically a basal level of expression even in the presence of a large concentration of repressor. We assume that this leakiness is also reflected by the fraction q such that a fraction of repressors less than q are active in the limit of infinite inducer concentration, with

(10) $$\begin{array}{r} p_{A}(c\to \infty )\approx \frac{{\left(\frac{c}{K_{A}}\right)}^{m}}{{\left(\frac{c}{K_{A}}\right)}^{m}+e^{-\beta \text{Δ}\epsilon }{\left(\frac{c}{K_{I}}\right)}^{m}}\\ =\frac{{\left(K_{I}\right)}^{m}}{{\left(K_{I}\right)}^{m}+e^{-\beta \text{Δ}\epsilon }{\left(K_{A}\right)}^{m}}\leq q. \end{array}$$

Rearranging Eqn. 10, we obtain a second condition

(11) $${\left(\frac{K_{A}}{K_{I}}\right)}^{m}\geq \frac{1-q}{q}e^{\beta \text{Δ}\epsilon }.$$

Eqns. 9 and 11 thus establish biologically reasonable bounds on the dissociation constants $K_{A,I}$ and the energy difference Δϵ for a given “error” q and number of binding sites m.

Suppose, for example, that less than 10% (q=0.1) of repressors are active in the presence of infinite inducer concentration. The parameters of this MWC model are then bounded by

(12) $$e^{-\beta \text{Δ}\epsilon }\leq \frac{1}{9}$$

and

(13) $${\left(\frac{K_{A}}{K_{I}}\right)}^{m}\geq 9e^{\beta \text{Δ}\epsilon }.$$

Fig. 3(B) highlights several viable probability curves within the bounded parameter regime defined by Eqns. 12 and 13. We assume throughout that there are m=2 binding sites for inducers on repressors. For fixed βΔϵ=4 and $K_{A}=150\;\mu M$, Fig. 3(B) then maps the probability of activity as a function of molar inducer concentration for increasing dissociation constant $K_{I}<K_{A}$. As the ratio $K_{d}^{A}/K_{d}^{I}$ decreases, approaching its lower bound (denoted in cyan in Fig. 3), the curve’s inflection point shifts toward higher inducer concentrations. This occurs because as $K_{d}^{I}$ increases and the inducer becomes less tightly bound to the inactive compared to the active repressor, a higher inducer concentration is necessary to sequester inactive repressors equivalently. Given that the choice of parameters in Fig. 3(A) is also within the biologically reasonable regime, we use these same parameters for the remainder of this work, unless otherwise stated.

Note that increasing the number of inducer binding sites m for the target repressor protein would increase the cooperativity of inducer-repressor interactions, and thus increase the sensitivity of response to the presence of inducer. This would make the slope of the probability function steeper at the inflection point. Keeping all other properties fixed, an increase in m also decreases the inducer concentration that is required to suppress repressor activity.

While this role of induction has been widely discussed and used in experimental literature, theoretical studies leveraging this perspective for parametrization remain ongoing, with recent work demonstrating the utility of induction in modeling the dynamics of simple gene regulatory motifs [57, 63]. In particular, the scope of this approach has not been expanded to the more intricate regulatory networks standard in nature. We now incorporate this definition for the probability of active repressor into our model for gene expression dynamics in the three-gene toggle switch.

### C. The baseline model as a function of inducer concentrations

Suppose now that each repressor produced from the three-gene toggle circuit in Fig. 1 can be controlled by its own inducer, each at concentrations $c_{1}$, $c_{2}$, and $c_{3}$. We will make the reasonable assumption that each repressor responds to induction with the same sensitivity and thus by the same probability function. As shown in Fig. 2(B), this means that expression of repressor 1 depends on the concentrations of active repressors 2 and 3, namely, $p_{\text{act}}\left(c_{2}\right)\times R_{2}$ and $p_{\text{act}}\left(c_{3}\right)\times R_{3}$. We similarly scale the regulatory contribution of $R_{1}$ to expression of $R_{2}$ and $R_{3}$ by the probability $p_{\text{act}}\left(c_{1}\right)$.

Eqns. 2 – 4 describing this system’s dynamics thus become

(14) $$\frac{dR_{1}}{dt}=\frac{a_{1}}{\left[1+{\left(\frac{p_{\text{act}}\left(c_{2}\right)R_{2}}{K_{12}}\right)}^{n}\right]\left[1+{\left(\frac{p_{\text{act}}\left(c_{3}\right)R_{3}}{K_{13}}\right)}^{n}\right]}-\frac{R_{1}}{\tau_{1}},$$

(15) $$\frac{dR_{2}}{dt}=\frac{a_{2}}{\left[1+{\left(\frac{p_{\text{act}}\left(c_{1}\right)R_{1}}{K_{21}}\right)}^{n}\right]\left[1+{\left(\frac{p_{\text{act}}\left(c_{3}\right)R_{3}}{K_{23}}\right)}^{n}\right]}-\frac{R_{2}}{\tau_{2}},$$

(16) $$\frac{dR_{3}}{dt}=\frac{a_{3}}{\left[1+{\left(\frac{p_{\text{act}}\left(c_{1}\right)R_{1}}{K_{31}}\right)}^{n}\right]\left[1+{\left(\frac{p_{\text{act}}\left(c_{2}\right)R_{2}}{K_{32}}\right)}^{n}\right]}-\frac{R_{3}}{\tau_{3}}.$$

We can simplify our model to a dimensionless form through several assumptions. First, we assume for analytic convenience that all repressors are produced (i.e., translated) and degraded at essentially the same maximal rates, independent of the underlying dynamics of transcription. This sets $a_{1}=a_{2}=a_{3}=a$ and $\tau_{1}=\tau_{2}=\tau_{3}=\tau$. We will also assume that a given repressor binds to different promoter sites with the same affinity, such that $K_{21}=K_{31}\equiv K_{1}$, $K_{12}=K_{32}\equiv K_{2}$, and $K_{13}=K_{23}\equiv K_{3}$. This allows us to re-express Eqns. 14 – 16 by transforming $\bar{t}=t/\tau$, ${\bar{R}}_{i}=R_{i}/K_{i}$, and ${\bar{a}}_{i}=\tau a/K_{i}$ from which we obtain the dimensionless equations

(17) $$\frac{d{\bar{R}}_{1}}{d\bar{t}}=\frac{{\bar{a}}_{1}}{\left[1+{\left(p_{\text{act}}\left(c_{2}\right){\bar{R}}_{2}\right)}^{n}\right]\left[1+{\left(p_{\text{act}}\left(c_{3}\right){\bar{R}}_{3}\right)}^{n}\right]}-{\bar{R}}_{1},$$

(18) $$\frac{d{\bar{R}}_{2}}{d\bar{t}}=\frac{{\bar{a}}_{2}}{\left[1+{\left(p_{\text{act}}\left(c_{1}\right){\bar{R}}_{1}\right)}^{n}\right]\left[1+{\left(p_{\text{act}}\left(c_{3}\right){\bar{R}}_{3}\right)}^{n}\right]}-{\bar{R}}_{2},$$

(19) $$\frac{d{\bar{R}}_{3}}{d\bar{t}}=\frac{{\bar{a}}_{3}}{\left[1+{\left(p_{\text{act}}\left(c_{1}\right){\bar{R}}_{1}\right)}^{n}\right]\left[1+{\left(p_{\text{act}}\left(c_{2}\right){\bar{R}}_{2}\right)}^{n}\right]}-{\bar{R}}_{3}.$$

Finally, for most of the discussion in this work we will simplify to a symmetric system in which all repressors bind at all sites with equal affinity such that $K_{1}=K_{2}=K_{3}=K$ (we will examine how breaking this symmetry affects shifts in dynamics in later sections). This assumption then means that ${\bar{a}}_{1}={\bar{a}}_{2}={\bar{a}}_{3}=\bar{a}$, and we arrive at the system of equations

(20) $$\frac{d{\bar{R}}_{1}}{d\bar{t}}=\frac{\bar{a}}{\left[1+{\left(p_{\text{act}}\left(c_{2}\right){\bar{R}}_{2}\right)}^{n}\right]\left[1+{\left(p_{\text{act}}\left(c_{3}\right){\bar{R}}_{3}\right)}^{n}\right]}-{\bar{R}}_{1},$$

(21) $$\frac{d{\bar{R}}_{2}}{d\bar{t}}=\frac{\bar{a}}{\left[1+{\left(p_{\text{act}}\left(c_{1}\right){\bar{R}}_{1}\right)}^{n}\right]\left[1+{\left(p_{\text{act}}\left(c_{3}\right){\bar{R}}_{3}\right)}^{n}\right]}-{\bar{R}}_{2},$$

(22) $$\frac{d{\bar{R}}_{3}}{d\bar{t}}=\frac{\bar{a}}{\left[1+{\left(p_{\text{act}}\left(c_{1}\right){\bar{R}}_{1}\right)}^{n}\right]\left[1+{\left(p_{\text{act}}\left(c_{2}\right){\bar{R}}_{2}\right)}^{n}\right]}-{\bar{R}}_{3}.$$

The original model defined by Eqns. 2 – 4 thus transforms from a high-dimensional parameter space as a function of inherent system properties to an at most three-dimensional parameter space defined by inducer concentrations $c_{1}$, $c_{2}$, and $c_{3}$ that can be directly controlled in experimental settings. Note that the simplifying assumptions introduced here are in no sense necessary and we could examine the much less symmetrical situations as well, but we find that this parameter choice makes the underlying dynamics most transparent.

## III. REGIMES OF MULTISTABILITY

For a given Hill exponent n and maximal (dimensionless) production rate $\bar{a}$, we now explore the dynamic profiles possible for the three-gene toggle switch as a function of inducer concentrations. In particular, we quantify the thresholds, or bifurcations, as a function of inducer that bring about fundamental shifts in dynamics. Our graphical analysis throughout this section will consider the large cooperativity case n=4 and choose $\bar{a}=2$. While we will also address how much the dynamic thresholds change with parameter choices, our focus in this section is specifically on the effects of induction at various levels. To this end, we begin by considering a system controlled only by a single inducer.

### A. Single inducer

Suppose a single inducer controls the dynamics of the three-gene toggle switch by regulating the activity of $R_{1}$. This means that $p_{\text{act}}\left(c_{1}\right)\equiv p_{\text{act}}(c)$ and $p_{\text{act}}\left(c_{2}\right)=p_{\text{act}}\left(c_{3}\right)=1$ such that ${\bar{R}}_{2}$ and ${\bar{R}}_{3}$ are always maximally active. Substituting these definitions into Eqns. 20 – 22, we obtain the single-inducer model

(23) $$\frac{d{\bar{R}}_{1}}{d\bar{t}}=\frac{\bar{a}}{\left(1+{\bar{R}}_{2}^{n}\right)\left(1+{\bar{R}}_{3}^{n}\right)}-{\bar{R}}_{1},$$

(24) $$\frac{d{\bar{R}}_{2}}{d\bar{t}}=\frac{\bar{a}}{\left[1+{\left(p_{\text{act}}(c){\bar{R}}_{1}\right)}^{n}\right]\left(1+{\bar{R}}_{3}^{n}\right)}-{\bar{R}}_{2},$$

(25) $$\frac{d{\bar{R}}_{3}}{d\bar{t}}=\frac{\bar{a}}{\left[1+{\left(p_{\text{act}}(c){\bar{R}}_{1}\right)}^{n}\right]\left(1+{\bar{R}}_{2}^{n}\right)}-{\bar{R}}_{3}.$$

For a given inducer concentration c, one then sets the above equations to zero and solves to obtain the possible steady state expressions.

We can first gain some analytical insight from these equations by considering certain limit cases, specifically those regarding the existence of single-repressor-dominant steady states, as a function of inducer concentration.

Consider the existence of an ${\bar{R}}_{2}$-dominant steady state whereby ${\bar{R}}_{1}={\bar{R}}_{3}=\varepsilon$ such that ${\bar{R}}_{1}^{n}$, ${\bar{R}}_{3}^{n}\to 0$. It follows from setting Eqn. 24 to zero that such a steady-state requires ${\bar{R}}_{2}=\bar{a}$. By similarly setting Eqns. 23 and 25 to zero, it follows that ${\bar{R}}_{1,3}=\bar{a}/\left(1+{\bar{R}}_{2}^{n}\right)$. We can therefore conclude that the system always has the following steady state regardless of inducer concentration:

(26) $${\bar{R}}_{ss}=\left({\bar{R}}_{1},{\bar{R}}_{2},{\bar{R}}_{3}\right)=\left(\frac{\bar{a}}{1+{\bar{a}}^{n}},\bar{a},\frac{\bar{a}}{1+{\bar{a}}^{n}}\right)$$

Similar logic for the existence of an ${\bar{R}}_{3}$-dominant state leads to a similar predicted steady state

(27) $${\bar{R}}_{ss}=\left({\bar{R}}_{1},{\bar{R}}_{2},{\bar{R}}_{3}\right)=\left(\frac{\bar{a}}{1+{\bar{a}}^{n}},\frac{\bar{a}}{1+{\bar{a}}^{n}},\bar{a}\right).$$

The conditions for an ${\bar{R}}_{1}$-dominant steady state, where ${\bar{R}}_{2}^{n}$, ${\bar{R}}_{3}^{n}\to 0$, follow from the same logic in Eqns. 23 – 25 as ${\bar{R}}_{1}=\bar{R}$ and

(28) $${\bar{R}}_{2}={\bar{R}}_{3}=\frac{\bar{a}}{1+{\left(p_{\text{act}}(c){\bar{R}}_{1}\right)}^{n}}.$$

Unlike the ${\bar{R}}_{2}$ and ${\bar{R}}_{3}$-dominant states in Eqns. 26 and 27, Eqn. 28 indicates that for the ${\bar{R}}_{1}$-dominant steady state the concentrations of non-induced repressors ${\bar{R}}_{2}$ and ${\bar{R}}_{3}$ depend on inducer concentration c. As c increases and the probability of activity decreases, the corresponding repressor concentrations at steady state increase. This also means, however, that at a sufficiently high inducer concentration, ${\bar{R}}_{2}^{n}$, ${\bar{R}}_{3}^{n}\to 0$ can no longer hold true and the steady state can no longer exist. Assuming that ${\bar{R}}_{2}={\bar{R}}_{3}\leq \epsilon$ satisfies the specified condition, we can derive the inducer bifurcation threshold by substituting this inequality into Eqn. 28 and rearranging, leading to the result

(29) $$p_{\text{act}}(c)\geq \frac{1}{\bar{a}}\sqrt[{n}]{\frac{\bar{a}}{\varepsilon }-1}.$$

At equality, the probability of Eqn. 29 thus represents a bifurcation threshold, where for lower probabilities (i.e, higher inducer concentrations) the system can no longer exist at an ${\bar{R}}_{1}$-dominant steady state.

The results in Eqns. 26 – 29 therefore indicate that at low inducer concentration the system is able to arrive at a steady state in which any gene amongst the three can dominate expression. When the probability of activity drops below the threshold analytically approximated in Eqn. 29, however, the presence of inducer is sufficient to suppress ${\bar{R}}_{1}$ activity, and the system can thus no longer maintain a steady state with ${\bar{R}}_{1}$-dominant expression. The sensitivity of ${\bar{R}}_{1}$ activity to tuning then depends on cooperativity (as approximated by Hill coefficient n) and on the protein expression rate $\bar{a}$.

**FIG. 4:**
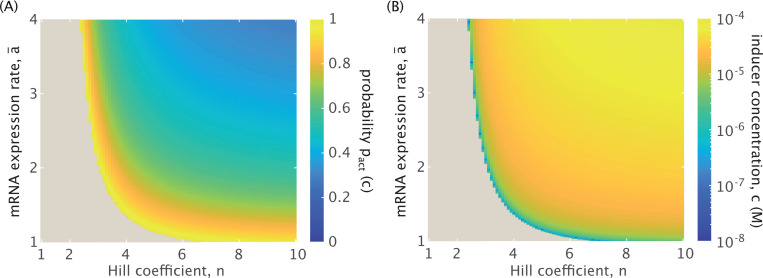
Heatmaps tracking the bifurcation threshold beyond which the three-gene toggle switch can no longer have an ${\bar{R}}_{1}$-dominant steady state. Note that we have defined the limit steady state case here as satisfying ${\bar{R}}_{2}^{n}$, ${\bar{R}}_{3}^{n}\to 0$, with ${\bar{R}}_{2}={\bar{R}}_{3}\leq \varepsilon$. The results shown are specific to the choice $\varepsilon =\sqrt[{n}]{0.01}$, i.e., the boundary for allowing tristable dynamics to be possible (A) The threshold probability of active repressor as a function of Hill coefficient n and mRNA expression rate $\bar{a}$. Note that the beige shaded region for smaller axis values denotes a regime in which the steady state is never possible at any inducer concentration. (B) The threshold in terms of inducer concentration for the MWC model as specified in Fig. 3(A).

Fig. 4(A) plots the probability threshold of Eqn. 29 as a function of n and $\bar{a}$. Note that the beige region of the heatmap depicts a regime where an ${\bar{R}}_{1}$-dominant steady state is not possible at any inducer concentration. The threshold for existence relies on sufficient cooperativity. Note, though, that when $\bar{a}≳2$, increasing the expression rate has little effect on this cooperativity bound. Fig. 4(B) plots the same relationship between the parameters, now explicitly as a function of inducer concentration (from the probability definition of Eqn. 6 in Section IIB).

The figure tracks cooperativities up to n=10. While it is not common for known multimeric transcription factors to extend beyond tetramers, plotting higher n acknowledges the uncertainty that remains in the field regarding the cooperative mechanics of eukaryotic regulation and particularly of enhancers [64]. Indeed, there are emerging cases of eukaryotic transcription factors functioning as higher-order homomultimeric complexes, such as FOXP3 in regulatory T-cells [65]. It is therefore useful to observe whether such putative higher-order cooperativities would influence bifurcations observed in the system.

We observe from the figure that the bifurcation threshold for this dynamic shift is most sensitive to parameter changes at the onset of the expression domain (i.e., at minimum required n and $\bar{a}$). For n≳4 and $\bar{a}≳2$, both parameters are sufficiently high that increasing either has little effect on the threshold probability and corresponding inducer concentration. It is therefore reasonable that, to understand the complexity of dynamics possible for the toggle switch, we choose n=4 as representative of dynamics in the highly-cooperative regime of the system for the remainder of this work. This is also a biologically reasonable choice, given the existence of homotetrameric transcription factors such as p53 that are crucial to cell fate decisions [66–68].

To show more completely how the dynamics evolve with increasing inducer concentration, we now choose a specific set of parameters (in this case from the highly cooperative regime of n=4 and $\bar{a}=2$) for Eqns. 23 – 25. At each inducer concentration, we numerically solve for the fixed point repressor concentrations of these equations. We then determine the stability of each fixed point by performing a linear stability analysis. We achieve this by evaluating the Jacobian which, for our three-gene network modeled by differential equations of the form $d{\bar{R}}_{i}/dt=f_{i}\left({\bar{R}}_{1},{\bar{R}}_{2},{\bar{R}}_{3}\right)$, is a 3 × 3 matrix with entries

(30) $$J_{ij}=\frac{\partial f_{i}}{\partial {\bar{R}}_{j}}.$$

The Jacobian matrix provides a first-order linear approximation of behavior evaluated near a fixed point, with its eigenvectors specifying the primary directions of dynamical motion from the fixed point, and with its eigenvalues reflecting the rates at which perturbations to the fixed point grow or decay along the corresponding eigenvectors. If all eigenvalues have negative real parts, perturbation in any eigenvector direction of expression space will decay back to the fixed point, indicating it to be a stable equilibrium. If any of the eigenvalues have a positive real part, however, perturbation in the corresponding eigenvector direction(s) will grow exponentially and the system will move away from the fixed point, marking it as unstable.

For the three-gene models examined throughout this study, the unstable fixed points observed are characterized as saddle points. Mathematically, this distinction arises because at least one eigenvalue is negative. Dynamic trajectories local to the fixed point thus move away in some dimensional directions (positive eigenvalues) and approach in others (negative eigenvalues). The number of positive eigenvalues determines the number of dimensional directions of “escape” from the fixed point and thus the “index” of the saddle. If we consider the dynamics of protein expression within a potential landscape, expression levels local to the highest index saddle essentially follow a hierarchical dynamic flow through expression space toward stable states, guided by the presence of saddle points with decreasing index.

**FIG. 5:**
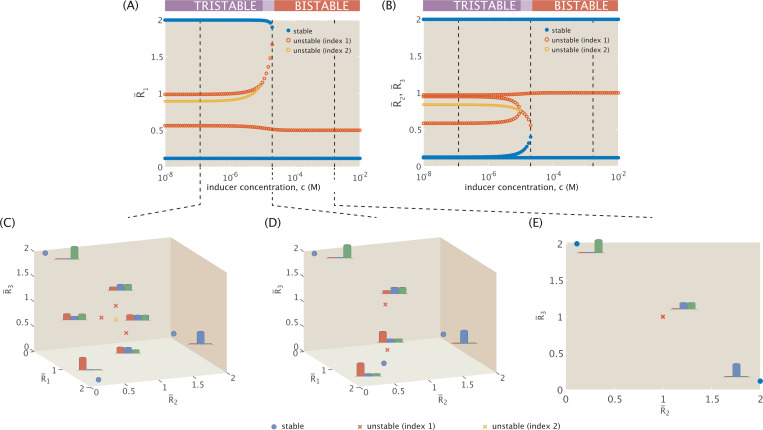
Dynamics of the three-gene toggle switch with n=4 and $\bar{a}=2$ for increasing inducer concentration c. Allosteric regulation is defined by the probability curve shown in Fig. 3(A). (A)-(B) Bifurcation diagrams for ${\bar{R}}_{1}$, ${\bar{R}}_{2}$, and ${\bar{R}}_{3}$ steady state expression as a function of inducer concentration. (C) Fixed points for the three-gene toggle switch at a low inducer concentration. (D) Fixed points for the three-gene toggle switch at an intermediate inducer concentration. (E) Fixed points for the three-gene toggle switch at a high inducer concentration. In (C)-(E) the stability of each fixed point is color-coded as in panels (A) and (B), with the expression levels for each fixed point colored as in Fig. 1.

Fig. 5 plots the resulting changes in the number of fixed points and their expression levels through bifurcation diagrams, isolating the trajectories of the ${\bar{R}}_{1}$ coordinate of fixed points in Panel (A), and the (overlapping) ${\bar{R}}_{2}$ and ${\bar{R}}_{3}$ coordinate fixed point trajectories in Panel (B). Three types of fixed points emerge from analysis of the Jacobian. Stable equilibria are denoted in blue, unstable index-1 saddle points in red, and unstable index-2 saddles in yellow.

At an intermediate inducer concentration, a saddle bifurcation occurs when a pair of fixed points annihilate each other. The intermediate regime, represented in Fig. 5(D), is still tristable, but the three stable states are now only connected by two saddle points. From an initial state with sufficient concentrations of all three repressors, the system can thus evolve dynamically in one of two ways: by either falling into a bistable potential well favoring ${\bar{R}}_{2}$ or ${\bar{R}}_{3}$ expression, or by following a trajectory directly stabilizing toward favoring ${\bar{R}}_{1}$ expression. Dynamics are therefore more restricted in this intermediate regime than in the low inducer regime, and no longer equally likely to evolve toward any given stable state.

Finally, Fig. 5(E) shows fixed point expression levels at a high inducer concentration, where the presence of inducer sufficiently suppresses the activity of ${\bar{R}}_{1}$, and the system undergoes a saddle bifurcation to collapse down to a bistable switch between ${\bar{R}}_{2}$ and ${\bar{R}}_{3}$-dominant expression.

Fig. 5 thus allows us to determine the inducer concentration thresholds at which dynamic shifts occur, including the shift between tristable and bistable dynamics. Given a distribution of stable state expression levels in the data, we could then distinguish between systems in the low inducer regime or in the more dynamically restricted intermediate regime. Fig. 5(B) also highlights that it is only at intermediate inducer concentrations that the system does not necessarily stabilize to a state exclusively favoring a single gene’s expression, which uniquely distinguishes the low vs. intermediate inducer concentration regimes of tristability.

**FIG. 6:**
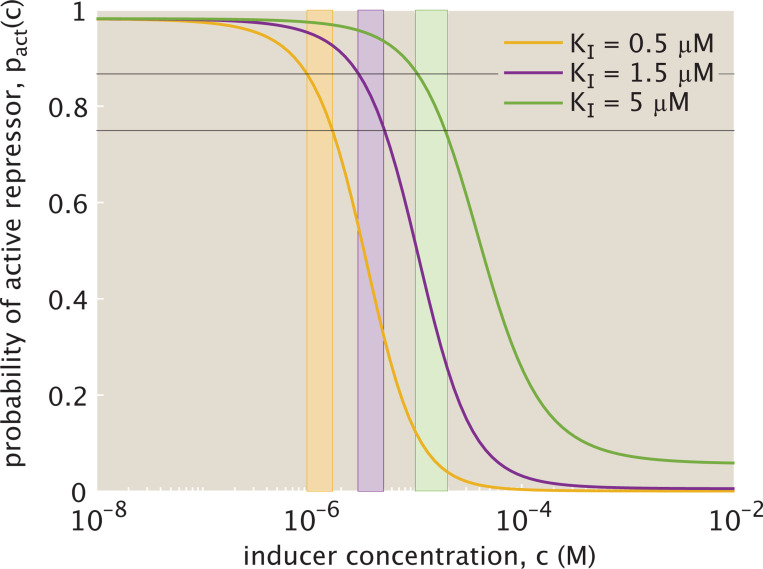
Change in inducer concentration range (shaded region) of the intermediate dynamic regime for increasing dissociation constant $K_{I}$, overlaid with the corresponding activity probability curves from Fig. 3. Note that regardless of $K_{I}$ parametrization the intermediate regime is found within the same range of probabilities $0.749\leq p_{\text{act}}(c)\leq 0.867$ (bounded in grey).

Given the relatively narrow concentration range for the intermediate dynamic regime, it is natural to ask how sensitive this window is to changes in allosteric interaction and to changes in the cooperativity, or ultrasensitivity, of expression response to induction. Regarding sensitivity to allostery, altering the ratio of $K_{A}$ and $K_{I}$ for an inducer binding to a repressor shifts the inflection point of the probability curve for activity toward higher inducer concentrations, as previously seen in Fig. 3. We thus observe in Fig. 6 that the log scale range of inducer concentrations falling within the intermediate dynamic regime does not change but shifts toward increasing value ranges with increasing $K_{I}$. Since altering the ratio of the dissociation constants does not affect the slope of the probability curve at the inflection point, the intermediate dynamic regime (for otherwise fixed values of m and Δϵ) exists consistently across different $K_{A}/K_{I}$ for $0.749\leq p_{\text{act}}(c)\leq 0.867$.

**FIG. 7:**
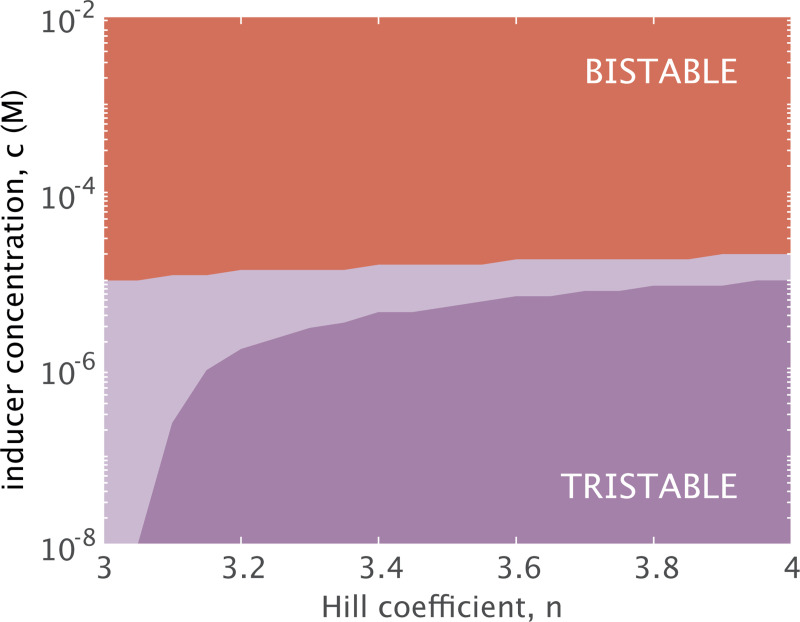
Change in the regions of multistability for n∈[3,4]. Each dynamic region is color-coded as denoted in Fig. 5, with the seven-fixed point tristable regime (purple), five-fixed point tristable regime (light purple), and three-fixed point bistable regime (red). Note that for approximately n≤3 the most complex dynamic regime is the light purple tristable regime with five fixed points.

The size of the intermediate regime may more likely change with a decrease in cooperativity. Fig. 4(B) indicates that at $\bar{a}=2$, bistability is possible for n>3, but the threshold for shifting from tristability to bistability shifts toward lower inducer concentrations for n<4. It is thus unclear from Fig. 4 alone whether the range of inducer concentrations allowing the intermediate dynamic regime would expand, contract, or remain the same for a smaller cooperative Hill coefficient. Indeed, Fig. 7 demonstrates how the regimes of multistability evolve in response to varying cooperativity. As the Hill coefficient increases from n=3 (the bound above which the system can exist in one of three possible dynamic regimes), the inducer concentration at which the system bifurcates from tristable to bistable dynamics rises but not significantly when viewed in the log scale. The threshold separating the two distinct regimes of tristability, however, does significantly change. The inducer concentration range corresponding to the intermediate regime diminishes with increasing cooperativity, since increased cooperativity means a more sensitive response in gene expression to the binding of repressors that emphasizes the bias away from ${\bar{R}}_{1}$ expression with increased induction. The tristable threshold begins to approach an apparent asymptote at n=4, confirming our earlier finding that increasing cooperativity to n=4 has little effect on the dynamics.

### B. Two inducers

Activity in gene regulatory networks is not always restricted to control from a single effector. Our framework allows for induction by multiple effectors, whether synthetically through additional experimentally-inputted non-metabolizable inducers [34], or through naturally-occurring coordinated inducer activity. The direct involvement of small molecule effectors is particularly important, for example, in metabolic sugar-inducible operons. Two effectors coordinate expression in these operons – the nucleotide messenger cAMP (with its concentration determined by the presence of glucose), and a carbon source [35, 69, 70]. For other natural inducer pairs, such as in cell differentiation, we are still learning how these effectors actually induce cell fates [16], and this motivates us to consider how dynamics in our three-gene toggle switch evolve in the presence of two inducers. We specifically extend to a system in which two repressors ${\bar{R}}_{1}$ and ${\bar{R}}_{2}$ are controlled by inducer concentrations $c_{1}$ and $c_{2}$, such that dynamics evolve by the differential equations

(31) $$\frac{d{\bar{R}}_{1}}{d\bar{t}}=\frac{\bar{a}}{\left(1+p_{\text{act}}\left(c_{2}\right){\bar{R}}_{2}^{n}\right)\left(1+{\bar{R}}_{3}^{n}\right)}-{\bar{R}}_{1},$$

(32) $$\frac{d{\bar{R}}_{2}}{d\bar{t}}=\frac{\bar{a}}{\left[1+{\left(p_{\text{act}}\left(c_{1}\right){\bar{R}}_{1}\right)}^{n}\right]\left(1+{\bar{R}}_{3}^{n}\right)}-{\bar{R}}_{2},$$

(33) $$\frac{d{\bar{R}}_{3}}{d\bar{t}}=\frac{\bar{a}}{\left[1+{\left(p_{\text{act}}\left(c_{1}\right){\bar{R}}_{1}\right)}^{n}\right]\left(1+p_{\text{act}}\left(c_{2}\right){\bar{R}}_{2}^{n}\right)}-{\bar{R}}_{3}.$$

**FIG. 8:**
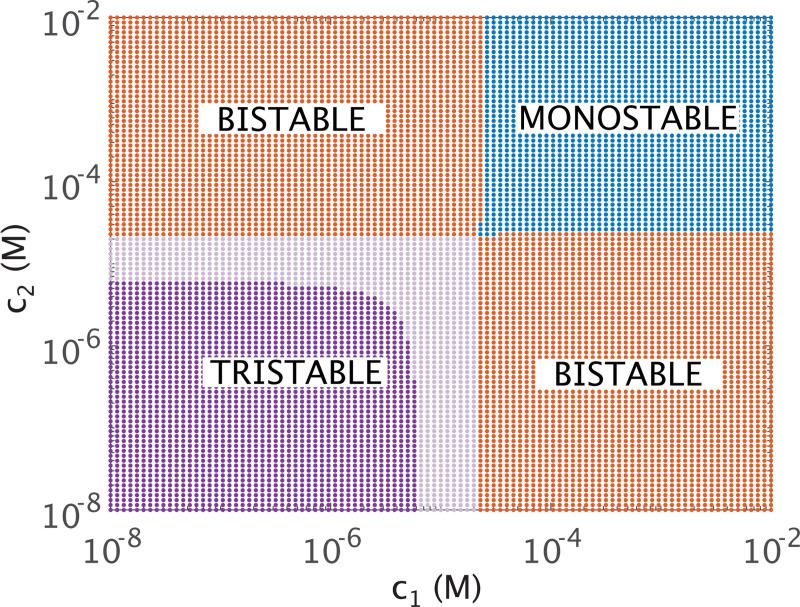
Phase diagram for evolving inducer concentrations $c_{1}$ and $c_{2}$ (regulating activity of ${\bar{R}}_{1}$ and ${\bar{R}}_{2}$, respectively). Each color-coded region corresponds to a phase defined by a different number of fixed points (seven in dark purple, five in light purple, three in red, and one in blue). Both inducers obey Eqn. 6 for the probability of activity with the same fixed parameters m=2, $\epsilon =4k_{B}T$, $K_{A}=150\;\mu M$, and $K_{I}=5\;\mu M$.

Fig. 8 plots the shifts in dynamic regions of phase space (color-coded by the number of fixed points) for inducer concentrations $c_{1}$ and $c_{2}$. Sufficiently high concentrations of both inducers render the target repressors largely inactive, such that the system enters a monostable regime (plotted in blue) that stabilizes to a single point reflecting expression of only the remaining non-induced repressor.

The bifurcation line seen at $p_{A}(c)\approx 0.725$ in Fig. 5 for one dimension now becomes a set of two linear thresholds intersecting at approximate inducer concentration $2.3\times 10^{-5}M$ (i.e., the approximate probability $p_{A}\left(c_{1}\right)=p_{A}\left(c_{2}\right)=0.7$. This intersection defines four regions with one (monostability in blue), two (bistability in red), or three stable points. Note that analytically we can show that the thresholds separating tristable from bistable dynamics for $c_{1}$ and $c_{2}$ are equal to those in Eqn. 29 (see Appendix A). In the purple region of Fig. 8, all three stable points are most dynamically accessible as both induction probabilities approach 1. Note that when the probability of active repressor reaches 1 for one of the repressors, the transition points along the axis of the other repressor reflect the bifurcations for the single-inducer system of Fig. 5, as expected. Increasing $K_{d}^{I}$ for one of the inducers would shift the curve so that the intersection of its asymptote with the corresponding inducer axis occurs at a higher concentration. This trend matches what we expect from the single inducer analysis.

The results observed for one and two inducers extend naturally in the three inducer regime, with tristability when all inducer concentrations are sufficiently small, monostability when all are sufficiently large, and bistability elsewhere. Similarly to how bifurcations transformed into a linear curve in two-dimensional phase space when moving from one to two regulating inducers, as in Fig. 8, the three inducer case likewise extends these curves into corresponding bifurcation planes in three-dimensions.

## IV. EFFECTS OF INDUCTION IN SYSTEMS PERTURBED AWAY FROM SYMMETRY

So far, we have assumed for simplicity that a given repressor binds different promoter sites with the same affinity (for example, ${\bar{R}}_{1}$ binds the promoters for ${\bar{R}}_{2}$ and ${\bar{R}}_{3}$ expression with the same affinity such that $K_{21}=K_{31}\equiv K_{1}$), and that protein expression is repressed with equal strengths $K_{1}=K_{2}=K_{3}\equiv K$. While this symmetric setting allows us to observe the evolution of complex bifurcation diagrams, the fact remains that many biological systems are naturally perturbed away from symmetry. It is therefore useful to introduce biases into the relative repression strengths of a given protein, defined such that the model can still take a dimensionless form using the same transformations that generated Eqns. 17 – 19 and Eqns. 20 – 22. This allows us to determine the sensitivity of the bifurcation thresholds to various perturbations.

Returning to the single-inducer case of Section IIIA, suppose that we keep all parameters fixed but introduce bias into regulation by the inducer-targeted ${\bar{R}}_{1}$. ${\bar{R}}_{1}$ regulates ${\bar{R}}_{2}$ and ${\bar{R}}_{3}$ expressions by binding to the respective promoter sites with affinities $K_{21}$ and $K_{31}$, respectively. We now let $K_{31}/K_{21}=\alpha$, with $K_{21}$ and all remaining dissociation constants equal to K as before. The dynamics are then defined by the differential equations

(34) $$\frac{d{\bar{R}}_{1}}{d\bar{t}}=\frac{\bar{a}}{\left(1+{\bar{R}}_{2}^{n}\right)\left(1+{\bar{R}}_{3}^{n}\right)}-{\bar{R}}_{1},$$

(35) $$\frac{d{\bar{R}}_{2}}{d\bar{t}}=\frac{\bar{a}}{\left[1+{\left(p_{\text{act}}(c){\bar{R}}_{1}\right)}^{n}\right]\left(1+{\bar{R}}_{3}^{n}\right)}-{\bar{R}}_{2},$$

(36) $$\frac{d{\bar{R}}_{3}}{d\bar{t}}=\frac{\bar{a}}{\left[1+{\left(\frac{p_{\text{act}}(c)}{\alpha }{\bar{R}}_{1}\right)}^{n}\right]\left(1+{\bar{R}}_{2}^{n}\right)}-{\bar{R}}_{3}.$$

**FIG. 9:**
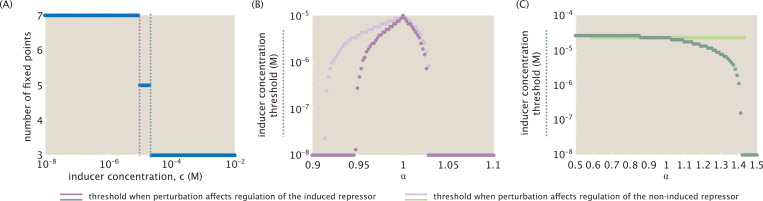
The change in bifurcation thresholds in a single inducer system as α rises. (A) The number of fixed points as a function of inducer concentration. The purple threshold denotes the transition between distinct tristable dynamic regimes (i.e., seven or five fixed points), and the green threshold denotes the transition between tristable and bistable dynamics (five or three fixed points, respectively). The system corresponds to that analyzed in Section IIIA, with α=1. (B) The change in the inducer concentration threshold at which the tristable (purple) transition occurs as a function of α. The dark purple curve corresponds to a system where α targets regulation of the induced repressor, ${\bar{R}}_{1}$. The light purple curve is for a system where α affects regulation of a non-induced repressor, in this case ${\bar{R}}_{2}$. (C) The change in the inducer concentration defining the threshold separating tristable from bistable dynamics as a function of α. The specific colors again correspond to the different targets for perturbation bias, with dark green targeting the induced repressor and light green the non-induced repressor.

In Section IIIA, we observed three distinct dynamic regimes, with decreasing complexity (i.e., number of fixed points) as the inducer concentration rose. Fig. 9(A) plots these shifts in dynamic regimes explicitly as changes in the number of fixed points, with the purple threshold denoting the bifurcation between distinct tristable regimes, and the green threshold denoting the bifurcation between tristable and bistable dynamics. The system assumes equal interaction strengths among all repression-binding event types, i.e., α=1.

We now demonstrate how these bifurcations shift as we move away from symmetry. Fig. 9(B) reveals a narrow window of perturbation away from symmetry for which a seven-fixed-point regime exists. Expressed differently, the dark purple curve indicates a window of only 0.946≲α≲1.028 for which a system with sufficiently high concentrations of all three repressors is equally likely to stabilize to any of the three possible stable states. The dark green curve in Fig. 9(C) shows that the threshold separating tristable from bistable dynamics is little affected by small perturbations around α=1 and by more sizeable perturbations α<1, for which ${\bar{R}}_{1}$ has a weaker affinity for the ${\bar{R}}_{2}$ promoter than for the ${\bar{R}}_{3}$ promoter. Favoring affinity to ${\bar{R}}_{2}$ for α>1, however, begins to affect the threshold inducer concentration noticeably. At α≳1.42, the system becomes bistable regardless of inducer concentration.

Combining the information in dark purple and dark green in Fig. 9(B) and (C), we conclude that for α<0.95 and 1.028≲α≲1.42 only two dynamic regimes are possible (with the seven-fixed point regime no longer viable at any inducer concentration). The perturbation threshold beyond which tristability of any kind is no longer possible occurs at α≈1.42.

Alternatively, we can consider a perturbation bias that affects regulation by a repressor that is not targeted by an inducer, such as ${\bar{R}}_{2}$. In this case, setting $K_{32}/K_{12}=\alpha$ and all remaining coefficients (including $K_{12}$) to K, we obtain the equations

(37) $$\frac{d{\bar{R}}_{1}}{d\bar{t}}=\frac{\bar{a}}{\left(1+{\bar{R}}_{2}^{n}\right)\left(1+{\bar{R}}_{3}^{n}\right)}-{\bar{R}}_{1},$$

(38) $$\frac{d{\bar{R}}_{2}}{d\bar{t}}=\frac{\bar{a}}{\left[1+{\left(p_{A}(c){\bar{R}}_{1}\right)}^{n}\right]\left(1+{\bar{R}}_{3}^{n}\right)}-{\bar{R}}_{2},$$

(39) $$\frac{d{\bar{R}}_{3}}{d\bar{t}}=\frac{\bar{a}}{\left[1+{\left(p_{A}(c){\bar{R}}_{1}\right)}^{n}\right]\left[1+{\left(\frac{{\bar{R}}_{2}}{\alpha }\right)}^{n}\right]}-{\bar{R}}_{3}.$$

The light purple and green curves in Fig. 9(B) and (C) indicate the change in thresholds as α rises and alters the regulatory bias of ${\bar{R}}_{2}$. Fig. 9(B) shows that the change in this threshold as ${\bar{R}}_{2}$ increases from 1 (such that ${\bar{R}}_{2}$ has a stronger binding affinity for the promoter of the induced ${\bar{R}}_{1}$ than for ${\bar{R}}_{3}$) is comparable to the change observed in the system case described by Eqns. 34 – 36. Decreasing α to favor stronger binding to the promoter for the non-induced ${\bar{R}}_{3}$, however, dampens the system’s response to bias. The system denoted in light purple thus has a larger window of perturbation away from symmetry where the seven-fixed-point tristable regime is possible, with the window now favoring regulation of the non-induced over the induced repressor’s expression.

The light green curve in Fig. 9(C), on the other hand, indicates that perturbation bias aimed to affect regulation by the non-induced repressor ${\bar{R}}_{2}$ in fact has no effect on the threshold between tristability and bistability until α≳1.45. We conclude that, unlike the first case targeting the induced repressor, perturbations targeting a non-induced repressor only influence how complex the system’s tristable dynamics can become.

## V. THE THREE-GENE TOGGLE SWITCH WITH SELF-ACTIVATION

**FIG. 10:**
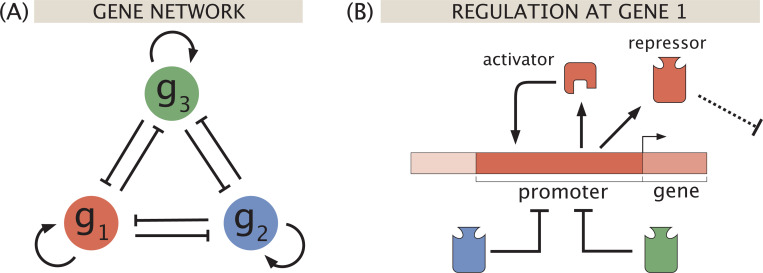
Gene expression in the three-gene toggle switch with self-activation. (A) Network of three mutually-repressing genes $g_{i}$, each producing a protein at average concentration $R_{i}$ that can either repress expression of $R_{j\neq i}$ or activate its own expression. (B) Regulatory binding and expression at the promoter site for gene 1. Note that a comparable illustration applies to genes 2 and 3.

The systems analyzed thus far focus exclusively on mutual repression. Systems exhibiting a bistable toggle switch, however, can also be modeled with gene products not only repressing other genes but also stimulating their own expressions through self-activation, as shown in Fig. 10(A). Fig. 10(B) highlights protein production and regulatory binding at a given promoter site (in this case for gene $g_{1}$), where the protein produced can act either as a repressor targeting other genes or as an activator of its own expression. These dual repression and self-activation capabilities are observed across a range of gene network motifs with varying complexity, including applications in the bacteriophage lambda switch [10, 12, 47], stem cell and developmental differentiation [16], and mammalian cell cycle progression [71]. We now consider the effect of self-activation on stability in the three-gene context.

The following sections assume, as in our previous baseline model from Section IIA, that different repressors can bind non-exclusively at a given gene promoter site to regulate expression. There are two possible ways, however, to incorporate activator binding. First, we analyze cases in which activators compete with repressors for the promoter such that an activator cannot bind if any repressor is bound, and vice versa. Section VA2 then explores non-exclusive binding, where both an activator and a repressor can bind simultaneously at a given promoter site such that repressors temper the effect of activation.

**FIG. 11:**
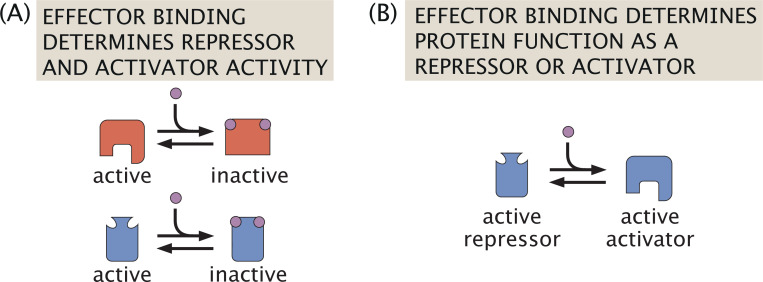
Two approaches to defining proteins as repressing or activating, depending on the function of effector binding. (A) Effector binding renders proteins inactive regardless of function because the binding site independently confers repressor vs. activator identity. (B) Effector binding directly determines whether a protein exists in a repressing or activating configuration, with a binding effect altering the protein from an active repressor to an active activator.

Given that all proteins in this self-activating network can act as either repressors or activators, we are interested not only in whether they bind competitively or non-exclusively to the DNA, but also in determining what defines a protein’s activity as a repressor or an activator. To do this, we highlight two biological mechanisms, and how protein activities in each case are tuned by distinct quantitative roles for effector binding. In Fig. 11(A), it is the binding site identity alone that determines a protein’s function as a repressor or activator. For example, if a protein binds to its own gene’s promoter site then it functions as an activator, and otherwise as a repressor. The activity of activator proteins is then defined by the same effector binding event that captures repressor activity, with both protein types rendered inactive by effector binding.

On the other hand, Fig. 11(B) presents a setting in which effector binding directly determines protein function. Since effector binding induces a protein conformational change (favoring expression in the inducer case), we propose in this case that effector binding alters a protein’s configuration from that of an active repressor to that of an active activator. Past precedent exists for modeling such effector-driven dual function transcription factors. For the arabinose operon, for example, the presence of arabinose induces a conformational change in AraC and thus determines its interaction with the DNA as either a repressor or an activator [72–74]. Effector molecules can also indirectly control transcription factor function by determining whether transcription factors recruit corepressors or coactivators when bound to the DNA, such as for steroid hormone receptors [75, 76].

We demonstrate in the following discussion how these different interpretations of effector-regulated activity lead to distinct dynamics for competitive repressor-activator binding and in particular for non-exclusive binding. In comparing the two types of mechanisms for effector activity, we continue to define thermodynamic states and weights for a system that does not explicitly involve long-range binding through DNA looping, although this could actually occur in nature. Models incorporating such looping have been considered in simpler single operon studies through grand canonical ensemble interpretations [54, 77], and could be adapted to our expanded structure and definition for induction. Our discussion here, however, will remain consistent with the analysis presented thus far, examining extensions of the bistable toggle switch to three genes with self-activation, and will focus specifically on the dynamics deriving specifically from our model of effector activity among repressors and activators.

### A. Competitive repressor-activator binding

**FIG. 12:**
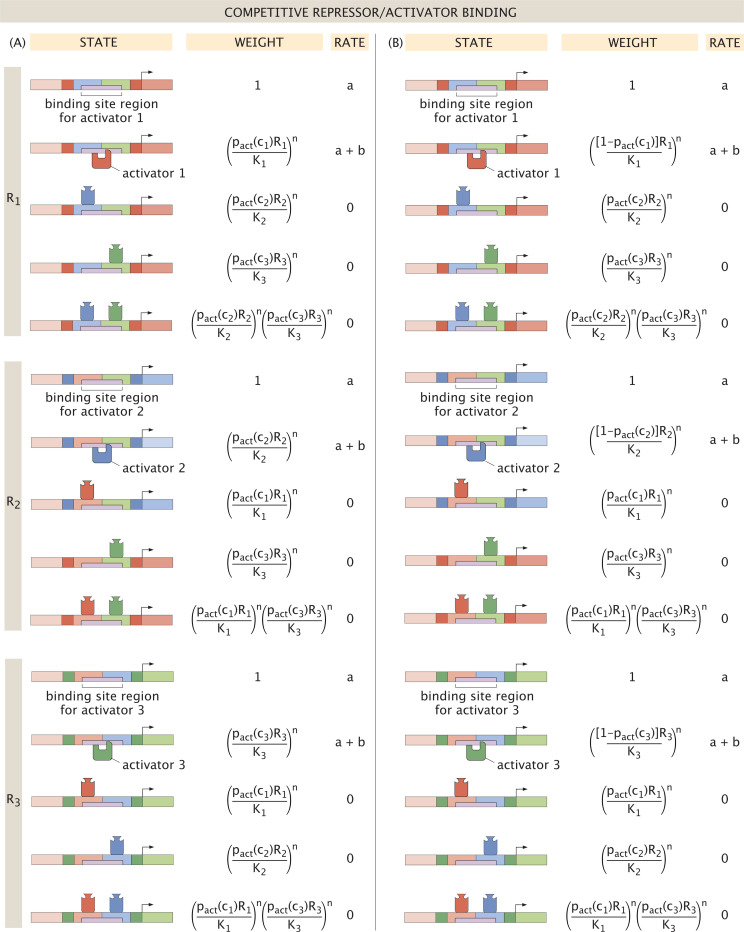
Expression of each protein $R_{i}$ in the three-gene toggle switch with self-activation and competitive binding for repressors and activators. (A) Competitive binding with the role of effectors defined as in Fig. 11(A). (B) Competitive binding with the role of effectors defined as in Fig. 11(B).

We begin by considering competitive binding, with thermodynamic states, weights, and rates as depicted in Fig. 12. If we first assume that the binding site determines a protein’s role as a repressor or an activator, then the activity of a given protein $R_{i}$ tuned by inducer concentration $c_{i}$ depends on the probability $p_{\text{act}}\left(c_{i}\right)$, as shown in Fig. 12(A). In line with the baseline model, a given protein is expressed at a maximal rate a in the absence of bound regulating proteins. A bound activator, however, now increases expression to a+b. From these states and weights, we then define the dynamics of protein expression by

(40) $$\frac{dR_{1}}{dt}=\frac{a+(a+b){\left(\frac{R_{1}}{K_{1}}\right)}^{n}}{1+{\left(\frac{R_{1}}{K_{1}}\right)}^{n}+{\left(\frac{R_{2}}{K_{2}}\right)}^{n}+{\left(\frac{R_{3}}{K_{3}}\right)}^{n}+{\left(\frac{R_{2}}{K_{2}}\right)}^{n}{\left(\frac{R_{3}}{K_{3}}\right)}^{n}}-\frac{R_{1}}{\tau },$$

(41) $$\frac{dR_{2}}{dt}=\frac{a+(a+b){\left(\frac{R_{2}}{K_{2}}\right)}^{n}}{1+{\left(\frac{R_{1}}{K_{1}}\right)}^{n}+{\left(\frac{R_{2}}{K_{2}}\right)}^{n}+{\left(\frac{R_{3}}{K_{3}}\right)}^{n}+{\left(\frac{R_{2}}{K_{2}}\right)}^{n}{\left(\frac{R_{3}}{K_{3}}\right)}^{n}}-\frac{R_{2}}{\tau },$$

(42) $$\frac{dR_{3}}{dt}=\frac{a+(a+b){\left(\frac{R_{3}}{K_{3}}\right)}^{n}}{1+{\left(\frac{R_{1}}{K_{1}}\right)}^{n}+{\left(\frac{R_{2}}{K_{2}}\right)}^{n}+{\left(\frac{R_{3}}{K_{3}}\right)}^{n}+{\left(\frac{R_{2}}{K_{2}}\right)}^{n}{\left(\frac{R_{3}}{K_{3}}\right)}^{n}}-\frac{R_{3}}{\tau }.$$

By transforming ${\bar{R}}_{i}=R_{i}/K_{1}$, $\bar{t}=t/\tau$, $\bar{a}=\tau a/K_{1}$, and $\bar{b}=\tau b/K_{1}$, we obtain the dimensionless form

(43) $$\frac{d{\bar{R}}_{1}}{d\bar{t}}=\frac{\bar{a}+(\bar{a}+\bar{b}){\bar{R}}_{1}^{n}}{1+{\bar{R}}_{1}^{n}+{\left(\frac{{\bar{R}}_{2}}{K^{\left(2\right)}}\right)}^{n}+{\left(\frac{{\bar{R}}_{3}}{K^{\left(3\right)}}\right)}^{n}+{\left(\frac{{\bar{R}}_{2}}{K^{\left(2\right)}}\right)}^{n}{\left(\frac{{\bar{R}}_{3}}{K^{\left(3\right)}}\right)}^{n}}-{\bar{R}}_{1},$$

(44) $$\frac{d{\bar{R}}_{2}}{d\bar{t}}=\frac{\bar{a}+(\bar{a}+\bar{b}){\left(\frac{{\bar{R}}_{2}}{K^{\left(2\right)}}\right)}^{n}}{1+{\bar{R}}_{1}^{n}+{\left(\frac{{\bar{R}}_{2}}{K^{\left(2\right)}}\right)}^{n}+{\left(\frac{{\bar{R}}_{3}}{K^{\left(3\right)}}\right)}^{n}+{\left(\frac{{\bar{R}}_{2}}{K^{\left(2\right)}}\right)}^{n}{\left(\frac{{\bar{R}}_{3}}{K^{\left(3\right)}}\right)}^{n}}-{\bar{R}}_{2},$$

(45) $$\frac{d{\bar{R}}_{3}}{d\bar{t}}=\frac{\bar{a}+(\bar{a}+\bar{b}){\left(\frac{{\bar{R}}_{3}}{K^{\left(3\right)}}\right)}^{n}}{1+{\bar{R}}_{1}^{n}+{\left(\frac{{\bar{R}}_{2}}{K^{\left(2\right)}}\right)}^{n}+{\left(\frac{{\bar{R}}_{3}}{K^{\left(3\right)}}\right)}^{n}+{\left(\frac{{\bar{R}}_{2}}{K^{\left(2\right)}}\right)}^{n}{\left(\frac{{\bar{R}}_{3}}{K^{\left(3\right)}}\right)}^{n}}-{\bar{R}}_{3},$$

where $K^{\left(2\right)}=K_{2}/K_{1}$ and $K^{\left(3\right)}=K_{3}/K_{1}$. For simplicity, we assume equal binding affinities such that $K^{\left(2\right)}=K^{\left(3\right)}=1$. We now examine how the system’s dynamic profile evolves with increasing inducer concentration, and how the increasing influence of activator binding toward expression affects the dynamic landscape.

#### 1. Induction via Case (A)

We introduce self-activation in the induced setting of “Case (A)” from Fig. 12(A) to observe how it alters the dynamics of the baseline model. Given the states and weights listed, and assuming $K^{\left(2\right)}=K^{\left(3\right)}=1$ and a single inducer c targeting ${\bar{R}}_{1}$, Eqns. 43 – 45 become

(46) $$\frac{d{\bar{R}}_{1}}{d\bar{t}}=\frac{\bar{a}+(\bar{a}+\bar{b}){\left[p_{\text{act}}(c){\bar{R}}_{1}\right]}^{n}}{1+{\left[p_{\text{act}}(c){\bar{R}}_{1}\right]}^{n}+{\bar{R}}_{2}^{n}+{\bar{R}}_{3}^{n}+{\bar{R}}_{2}^{n}{\bar{R}}_{3}^{n}}-{\bar{R}}_{1},$$

(47) $$\frac{d{\bar{R}}_{2}}{d\bar{t}}=\frac{\bar{a}+(\bar{a}+\bar{b}){\bar{R}}_{2}^{n}}{1+{\left[p_{\text{act}}(c){\bar{R}}_{1}\right]}^{n}+{\bar{R}}_{2}^{n}+{\bar{R}}_{3}^{n}+{\bar{R}}_{2}^{n}{\bar{R}}_{3}^{n}}-{\bar{R}}_{2},$$

(48) $$\frac{d{\bar{R}}_{3}}{d\bar{t}}=\frac{\bar{a}+(\bar{a}+\bar{b}){\bar{R}}_{3}^{n}}{1+{\left[p_{\text{act}}(c){\bar{R}}_{1}\right]}^{n}+{\bar{R}}_{2}^{n}+{\bar{R}}_{3}^{n}+{\bar{R}}_{2}^{n}{\bar{R}}_{3}^{n}}-{\bar{R}}_{3}.$$

To allow direct comparison to the original single-inducer model in Eqns. 23 – 25, where n=4 and $\bar{a}=2$, we again set n=4 and now choose maximal expression to satisfy $\bar{a}+\bar{b}=2$.

**FIG. 13:**
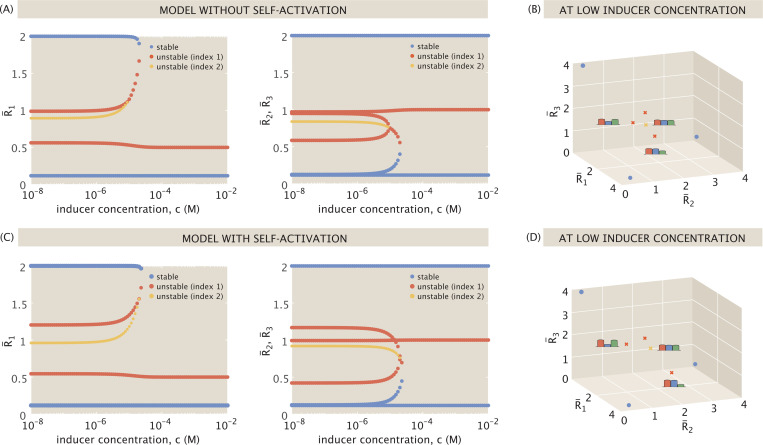
Comparison of the dynamics for the three-gene toggle switch without and with the presence of self-activation. In both settings, the Hill coefficient is n=4 and we set the same maximal production level. (A) Bifurcation diagrams for ${\bar{R}}_{1}$, ${\bar{R}}_{2}$, and ${\bar{R}}_{3}$ steady-state expressions without self-activation as a function of inducer concentration, as previously shown in Fig. 5(A). Maximal production is set at $\bar{a}=2$. (B) Fixed points at low inducer concentration. (C) Bifurcation diagrams for steady-state expression with self-activation as defined by Eqns. 43 – 45. Rates are set at $\bar{a}=2$ and $\bar{b}=0$ for maximal production $\bar{a}+\bar{b}=2$. (D) Fixed points at low inducer concentration.

Fig. 13 directly compares the dynamics that emerge. Panel (A) plots the bifurcations for the baseline setting previously shown in Fig. 5, with panel (B) highlighting the fixed points at low inducer concentration in three-dimensional expression space. Panel (C) plots the bifurcations for the comparable system with self-activation in Eqns. 46 – 48, with panel (D) again highlighting the low inducer regime. While any number of possible $\left(\bar{a},\bar{b}\right)$ pairs can satisfy $\bar{a}+\bar{b}=2$, for closest comparison, we choose the limit case of $\bar{a}=2$ and $\bar{b}=0$, at which weak activator binding has a negligible impact on expression.

Fig. 13(C) shows that self-activation does not affect stable expression levels or the system’s dynamics at high inducer concentrations. It does, however, influence the remaining saddle points, further accentuating expression of the dominant proteins. For example, the index-2 saddle becomes more centered in expression space at the half-maximal expression level. Also, for each index-1 saddle that includes ${\bar{R}}_{1}$ as a dominant protein, the dominant protein expression levels become more pronounced compared to those that are suppressed. This means that each saddle point allowing significant ${\bar{R}}_{1}$ expression is further from the stable points it dynamically links. If the system were to begin with half-maximal expression of each protein, for example, any trajectories leaning toward the ${\bar{R}}_{1}-{\bar{R}}_{2}$ or ${\bar{R}}_{1}-{\bar{R}}_{3}$ switches will take longer to stabilize because they must travel further to approach an index-1 saddle before veering off toward a stable point. We can thus think of these stable states as lying in deeper potential wells, making the related systems less likely to transition from one stable state to another.

**FIG. 14:**
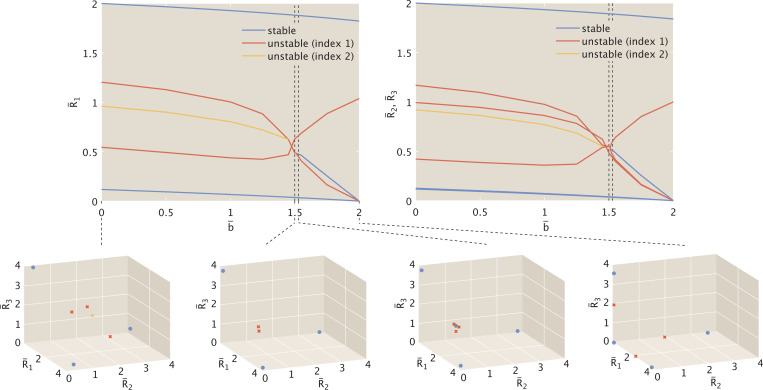
Bifurcation diagrams for ${\bar{R}}_{1}$, ${\bar{R}}_{2}$, and ${\bar{R}}_{3}$ expression at a low inducer concentration and at fixed $\bar{a}+\bar{b}=2$ as a function of increasing activating strength $\left(\bar{b}\right)$. The corresponding set of fixed points are shown in three-dimensional expression space beneath the bifurcation diagrams, specifically highlighting each distinct dynamic phase represented by $\left(\bar{a},\bar{b})=(2,0\right)$ (also shown in Fig. 13(B)), $\left(\bar{a},\bar{b})=(0.5,1.5\right)$, $\left(\bar{a},\bar{b})=(0.48,1.52\right)$, and $\left(\bar{a},\bar{b})=(0,2\right)$.

As one might expect, the differences between the baseline model and our chosen parametrization of the self-activation model are subtle, but the key property of self-activation is the further tunability possible through the additive expression effect from $\bar{b}$. Increasing the strength of activation while maintaining a constant maximal expression level $\bar{a}+\bar{b}$ can lead to significant dynamic transformations. Fig. 14 now fixes the system at a low inducer concentration for $\bar{a}+\bar{b}=2$, and plots the bifurcation diagram with dynamics tuned as a function of expression $\bar{b}$. As $\bar{b}$ increases from zero, all fixed points shift toward lower expression while retaining the typical dynamic profiles observed in Fig. 13. Plotting how the fixed points shown for $\left(\bar{a},\bar{b})=(2,0\right)$ would change with increasing inducer concentration, for example, would generate two dynamic phase shifts to arrive at a bistable switch between ${\bar{R}}_{2}$ and ${\bar{R}}_{3}$ expression, with results comparable to those shown in Fig. 5.

With sufficiently strong activation, however, at $\bar{b}\approx 1.47$ the system no longer retains the index-2 saddle point. This is evidenced by the fixed point plot highlighted at $\bar{b}=1.5$ in Fig. 14. Moreover, $\bar{b}=1.52$ marks a bifurcation threshold from tristable to *quadristable* expression, where the previously index-2 fixed point essentially transforms into a stable point. Rather than comprising of a set of switches between ${\bar{R}}_{i}$ and ${\bar{R}}_{j}$ expression, the high $\bar{b}$ system now brings together a set of switches between on or off expressions for each protein. Note that, as the bifurcation point, $\bar{b}=1.52$ shares properties between the two system phases it straddles. As shown in Fig. 14, it allows the quadristable dynamics observed for higher $\bar{b}$ at low inducer concentration, but still reduces to a single bistable switch at high inducer concentration (as is characteristic of the small $\bar{b}$ regime). Systems approaching the limit where expression can only occur in the presence of bound activator, on the other hand, as in the highlighted example $\left(\bar{a},\bar{b})=(0,2\right)$ of Fig. 14, reduce down to tristability at high inducer concentration, allowing either ${\bar{R}}_{2}$-dominant expression, ${\bar{R}}_{3}$-dominant expression, or none.

#### 2. Induction via Case (B)

We now compare Case (A) above to the dynamics arising in an alternative induction setting where the effector molecule binding determines whether a protein is active as a repressor or as an activator. Fig. 12(B) outlines the thermodynamic states and weights in this setting, from which we model the system with the differential equations

(49) $$\frac{d{\bar{R}}_{1}}{d\bar{t}}=\frac{\bar{a}+(\bar{a}+\bar{b}){\left[\left(1-p_{\text{act}}(c)\right){\bar{R}}_{1}\right]}^{n}}{1+{\left[\left(1-p_{\text{act}}(c)\right){\bar{R}}_{1}\right]}^{n}+{\bar{R}}_{2}^{n}+{\bar{R}}_{3}^{n}+{\bar{R}}_{2}^{n}{\bar{R}}_{3}^{n}}-{\bar{R}}_{1},$$

(50) $$\frac{d{\bar{R}}_{2}}{d\bar{t}}=\frac{\bar{a}+(\bar{a}+\bar{b}){\bar{R}}_{2}^{n}}{1+{\left[p_{\text{act}}(c){\bar{R}}_{1}\right]}^{n}+{\bar{R}}_{2}^{n}+{\bar{R}}_{3}^{n}+{\bar{R}}_{2}^{n}{\bar{R}}_{3}^{n}}-{\bar{R}}_{2},$$

(51) $$\frac{d{\bar{R}}_{3}}{d\bar{t}}=\frac{\bar{a}+(\bar{a}+\bar{b}){\bar{R}}_{3}^{n}}{1+{\left[p_{\text{act}}(c){\bar{R}}_{1}\right]}^{n}+{\bar{R}}_{2}^{n}+{\bar{R}}_{3}^{n}+{\bar{R}}_{2}^{n}{\bar{R}}_{3}^{n}}-{\bar{R}}_{3}.$$

For reasons that become clear once plotted, we consider systems allowing a larger maximal expression of $\bar{a}+\bar{b}=4$. Specifically, we will set $\bar{a}=\bar{b}=2$ for equally strong contributions from the basal and activated expression levels.

**FIG. 15:**
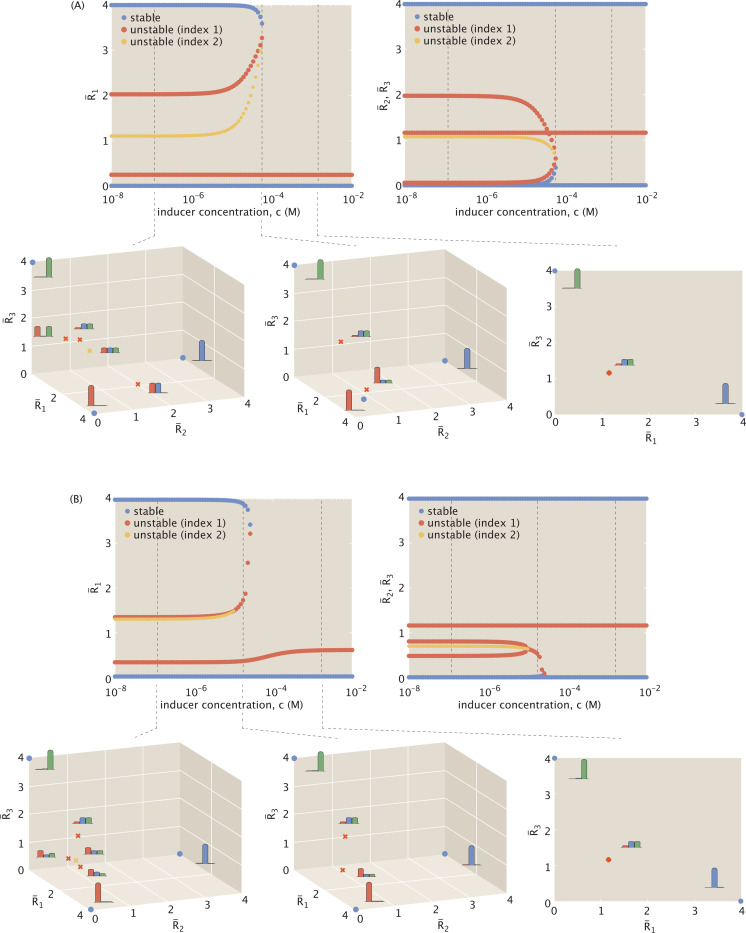
Induction cases (A) vs. (B) for competitive repressor-activator binding, with $\bar{a}=2$, $\bar{b}=2$. Each panel plots the bifurcation diagrams for ${\bar{R}}_{1}$, ${\bar{R}}_{2}$, and ${\bar{R}}_{3}$ steady-state expressions as a function of inducer concentration. The fixed points are visualized in gene expression space below these bifurcations at low, intermediate, and high inducer concentrations. The low and high inducer concentrations chosen are the same in (A) and (B), and the intermediate inducer concentrations selected highlight the same intermediate dynamic regime. (A) The competitive repressor-activator binding case in which inducer binding controls transcription factor activity regardless of its ultimate function as a repressor or activator. (B) The competitive repressor-activator binding case in which inducer binding transforms a protein from a repressing conformation to an activating form.

Fig. 15 compares the system defined by induction case (A), now for $\bar{a}=\bar{b}=2$, with the corresponding model in panel (B) for the alternative induction approach highlighted in Eqns. 49 – 51. We observe that in the low inducer concentration regime, while the dynamical structure remains the same, the saddle points allowing ${\bar{R}}_{1}$ expression are skewed toward favoring ${\bar{R}}_{1}$ expression. In this regime, a low inducer concentration means a high probability $p_{\text{act}}(c)$ of ${\bar{R}}_{1}$ acting as a repressor, and low probability $1-p_{\text{act}}(c)$ of acting as an activator. With $\left[1-p_{\text{act}}(c)\right]{\bar{R}}_{1}\to 0$ in Eqn. 49, ${\bar{R}}_{1}$ expression is essentially limited only by regulatory repression from ${\bar{R}}_{2}$ and ${\bar{R}}_{3}$, while the remaining two repressors are additionally regulatsed by $p_{\text{act}}(c){\bar{R}}_{1}$. The resulting skewness of thse central cluster to favor ${\bar{R}}_{1}$ places the ${\bar{R}}_{2}$ and ${\bar{R}}_{3}$-dominant stable states in deeper potential wells. It thus requires a less significant perturbation to transition out of the ${\bar{R}}_{1}$-dominant stable state into one of the other stable states.

Since all ${\bar{R}}_{1}$-expressing saddle points are spatially closer together in panel (B) compared to panel (A), this also means that case (B) of induction does not require as high an inducer concentration to transition to an intermediate dynamic regime. This also shifts the bifurcation that separates tristable from bistable dynamics to occur at a higher inducer concentration. When the inducer concentration is sufficiently high, the system transforms into one where ${\bar{R}}_{1}$ expression is effectively regulated by components beyond just ${\bar{R}}_{2}$ or ${\bar{R}}_{3}$. This shifts the saddle point toward a slightly higher ${\bar{R}}_{1}$ concentration, which serves to deepen the potential well between the ${\bar{R}}_{2}$ and ${\bar{R}}_{3}$-dominant stable states.

### B. Non-exclusive repressor-activator binding

We now turn to systems in which repressors and activators bind non-exclusively at a gene’s regulatory region. This means that at a given promoter site, a gene’s expression can be regulated not only by the presence of one or both repressors produced by the remaining two genes, but also by the possible additional presence of bound activator. As in the competitive binding case visualized in Fig. 12, this non-exclusive binding system retains the same possible states for a given promoter site, namely (i) the state with no bound transcription factors (with an expression rate a), (ii) states with one or both possible repressors bound (with an expression rate 0), and (iii) states with activator bound alone (with an expression rate a+b). Additional states, however, account for activated expression in the presence of one or both available repressors. In these states, activated expression is suppressed by the presence of bound repressor, defined by a corresponding rate a+b−d.

Upon deriving a set of differential equations in dimensionless form for the expression of proteins ${\bar{R}}_{1},{\bar{R}}_{2}$, and ${\bar{R}}_{3}$ from the corresponding thermodynamic states and weights (see Appendix B for details), we have, as in the competitive binding setting, a system defined by basal expression $\bar{a}$ and additional activated expression $\bar{b}$, with the maximal expression level constrained to $\bar{a}+\bar{b}=2$ to facilitate comparison across models. Given the non-exclusive binding conditions modeled here, expression now also depends on the strength of repression from $\bar{d}$, where $\bar{d}\leq \bar{a}+\bar{b}$.

The following subsections focus on the impact of effector concentration and the relative strengths of activation and repression on dynamic stability. This allows us to determine the range of dynamic landscapes possible within this parameter space under different interpretations for effector activity, and the sensitivity of the resulting dynamics and bifurcations to each of the rate parameters.

#### 1. Induction via Case (A)

We first consider Case (A) from Fig. 11, in which the binding of the effector molecule renders proteins inactive regardless of whether they are functioning as repressors or activators. Continuing our convention of choosing ${\bar{R}}_{1}$ as the target of effector binding, the concentration of ${\bar{R}}_{1}$ is thus scaled by the probability of activity $p_{\text{act}}(c)$, regardless of function, where we assume that all proteins interact with effectors through the same MWC model and binding affinities. Fig. 22 and Eqns. B5 - B7 of Appendix B define the corresponding states and weights along with the resulting dimensionless differential equations, incorporating the role of $p_{\text{act}}(c)$.

Evaluating the fixed points by numerical approximation, we find that allowing non-exclusive binding expands the possible set of stable states to the maximum number of combinatorial states, i.e., no expression, one gene expressing, two genes expressing, or all three expressing. Determining the likelihood of each state being available within the allowable rate parameter space offers insight into how this more complex regulatory architecture prioritizes expression.

**FIG. 16:**
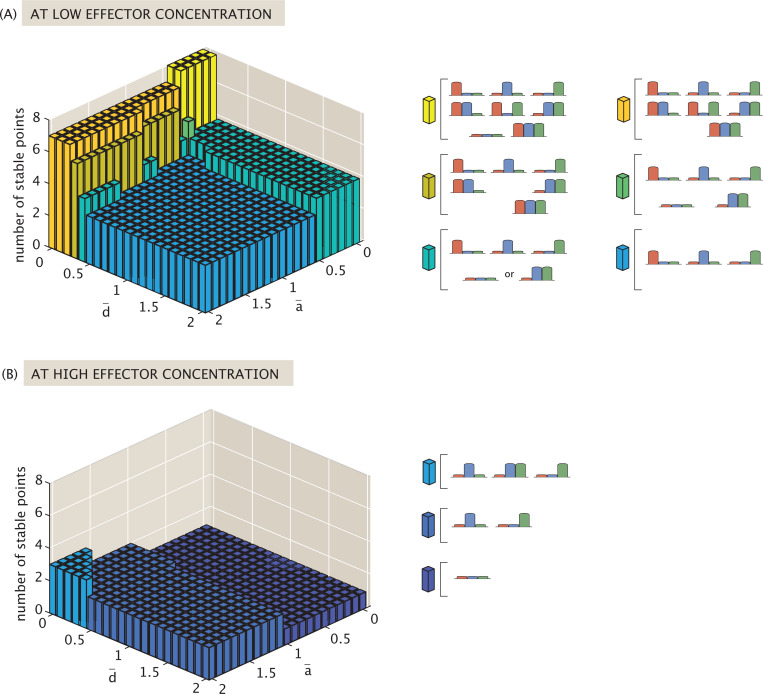
Number of stable fixed points in case (A) of non-exclusive repressor-activator binding. The plots highlight stable points at low (10^−8^ M) and high (10^−2^ M) effector concentrations in panels (A) and (B), respectively, for varying rate parameters $\bar{a}$, $\bar{b}$, and $\bar{d}$, where $\bar{a}+\bar{b}=2$ and $\bar{d}\leq \bar{a}+\bar{b}$. The diagrams on the right hand side of panel (A) represent the types of stable points observed in each regime at low effector concentration, i.e., with eight (yellow), seven (orange), six (brown), five (green), four (teal), or three (blue) stable points. The diagrams on the right side of panel (B) represent the types of stable points observed in each regime at high effector concentration, i.e., with three (blue), two (dark blue), or one (purple) stable point(s).

Fig. 16(A) tracks the number of possible stable states that exist at low effector concentration $\left(c=10^{-8}\;\text{M}\right)$. The maximum number of stable states occurs in the yellow region where $\bar{d}\approx 0$ and $\bar{a}<0.5\;(\bar{b}>1.5)$. This represents systems in which repressor binding has negligible or no effect on expression, and gene expression relies heavily on bound activators.

As $\bar{a}$ and $\bar{d}$ increase, the complexity of the dynamic landscape diminishes, with the number of possible stable points falling until $\bar{a}≳0.5$ and $\bar{d}≳0.5$. This threshold combination is sufficient to render the system tristable at low effector concentrations, and is similar to what we observed in the previous competitive binding models of Sections VA1 and VA2.

Beyond the three possible single-gene expression states, which exist at low effector concentrations for all possible rate parameter combinations, the stable state in which both ${\bar{R}}_{2}$ and ${\bar{R}}_{3}$ dominate survives across the broadest allowable parameter space. Such a state is no longer possible only if $\bar{a}≳0.5$ and $\bar{d}≳0.5$. The robustness of this state makes sense given that the model in question specifically tunes ${\bar{R}}_{1}$ expression via effector concentration. This targeted tuning thus more indirectly (and weakly) affects the existence of a stable $\left(0,{\bar{R}}_{2},{\bar{R}}_{3}\right)$ state. Fig. 16(A) also indicates that the repression parameter $\bar{d}$ drives the loss of the $\left({\bar{R}}_{1},0,{\bar{R}}_{3}\right)$ and $\left({\bar{R}}_{1},{\bar{R}}_{2},0\right)$ states in parameter space. Essentially, once repressors play an active role in regulation (no longer $\bar{d}\to 0$), dual repressor and activator activity implies that the system can only express ${\bar{R}}_{1}$ significantly in a stable state when it is the only protein being expressed.

Generally, as effector concentrations increase, the number of available stable fixed points decreases until the system reaches monostable, bistable, or tristable dynamics, as shown in Fig. 16(B). When $\bar{a}≲1$, such that activation contributes more strongly than basal expression, a high effector concentration suppresses all gene expression. When the system approaches the limit case of largely unregulated expression (i.e., $\bar{a}\to 2$ and $\bar{d}\to 0$) it is tristable, expressing either ${\bar{R}}_{2}$, ${\bar{R}}_{3}$, or both. Otherwise, in the relatively weak activation regime that remains, the system collapses to the bistable toggle switch between ${\bar{R}}_{2}$ and ${\bar{R}}_{3}$.

**FIG. 17:**
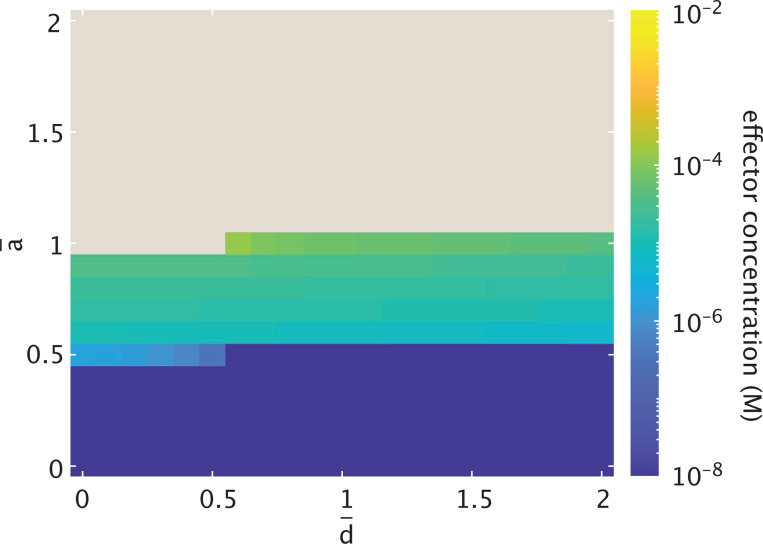
Heatmap tracking the minimum effector concentration (M) at which the stable state with no gene expression can exist for the system with non-exclusive activator-repressor binding (case (A)). Measurements span the range of rate parameters allowed by the constraints $\bar{a}+\bar{b}=2$ and $\bar{d}\leq \bar{a}+\bar{b}$, with the x-axis denoting increasing strength of repression $\bar{d}$, and the y-axis denoting decreasing strength of activation with increasing $\bar{a}$. Beige represents the parameter space in which a stable state without expression does not exist at any effector concentration.

**FIG. 18:**
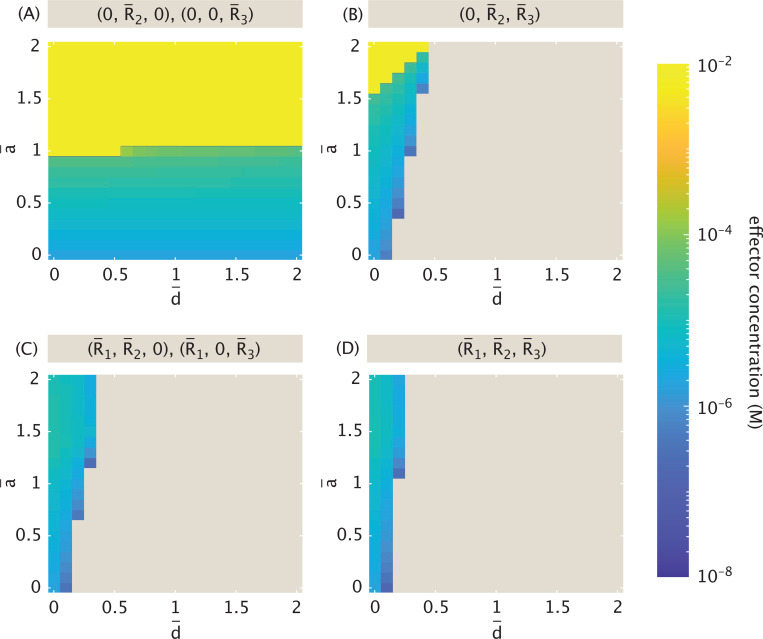
Heatmaps tracking the maximum effector concentrations (M) at which stable states can exist with the non-exclusive activator-repressor binding model described in Fig. 11(A). The measured values represent bifurcation thresholds for the existence of these states. The heatmaps track (A) stable states expressing one of the genes that does not directly interact with effector, i.e., $\left(0,{\bar{R}}_{2},0\right)$ and $\left(0,0,{\bar{R}}_{3}\right)$, (B) the stable state in which both genes not impacted by effector are expressed, i.e., $\left(0,{\bar{R}}_{2},{\bar{R}}_{3}\right)$, (C) stable states expressing two genes including the effector-controlled ${\bar{R}}_{1}$, and (D) the stable state expressing all three genes. Measurements span the range of rate parameters allowed by the constraints $\bar{a}+\bar{b}=2$ and $\bar{d}\leq \bar{a}+\bar{b}$, with the x-axis denoting increasing strength of repression $\bar{d}$, and the y-axis denoting decreasing strength of activation with increasing $\bar{a}$. Beige represents the region of parameter space in which the stable state cannot exist at any effector concentration, and yellow indicates that the stable state is possible at all effector concentrations

Figs. 17 and 18 illustrate further how the system evolves toward these different regimes as the effector concentration increases, and how sensitive the different stable states are to a change in effector concentration. These figures plot (respectively) the minimum and maximum effector concentrations necessary for different stable fixed points to exist, where Fig. 17 specifically highlights the minimum concentration for the stable state with no gene expression. At the maximum concentration threshold, a bifurcation occurs where a sufficiently high effector concentration prevents the system from stabilizing to the state with no expression. Beige denotes the part of parameter space where a given fixed point does not exist at any effector concentration, and yellow denotes the space where the fixed point survives at high effector concentration.

Notably, the heatmaps of Fig. 17 and Fig. 18(A) directly overlap, indicating a close relationship between the existence of a stable state in which no genes express, and the existence of a state that expresses one of the two genes not impacted by the effector ${\bar{R}}_{2}$ or ${\bar{R}}_{3}$). The existence of these three stable states is thus defined almost fully by the strength of activation as reflected by the parameter $\bar{a}$. As $\bar{a}$ rises, three regimes emerge with different dynamic trends for these stable states as the effector concentration increases. When $\bar{a}≲0.5$, all three states are possible until an intermediate effector concentration is reached, beyond which only the state suppressing all expression can exist among the three. When $0≲\bar{a}≲1$, we observe that a tradeoff occurs at an intermediate effector concentration, where the system’s ability to stabilize to an ${\bar{R}}_{2}$ or ${\bar{R}}_{3}$-dominant state at low concentrations is swapped for the system’s ability to suppress all expression. This indicates a regime of strong activation compared to basal expression in which an increase in effector concentration causes a fixed point to emerge and take the place of states that existed at lower concentrations. Finally, in the weak activation regime of $\bar{a}≳1$, the system can always stabilize to an ${\bar{R}}_{2}$ or ${\bar{R}}_{3}$-dominant state at all effector concentrations and never suppresses all expression. Note in all of these regimes that the strength of repression $\left(\bar{d}\right)$ has negligible or no effect on the existence of the three states.

Fig. 18(B)-(D) highlights stable fixed points where more than one type of protein is expressed. The existence of such points relies on weak repression $\left(\bar{d}<0.5\right)$. As $\bar{a}$ rises and the strength of activation decreases accordingly, we observe increases in the effector concentrations at which these stable points vanish. The heatmaps indicate that as repression strength rises, a higher value of $\bar{a}$ and thus a weaker degree of activation is needed for the system to stabilize to these states, with the state in (B) responding most gradually and the state in (D) most sharply as repression strength increases. Finally, the span of effector concentrations indicates that the bifurcation thresholds for these points are more responsive to changes in activation strength than those of the single-protein expression states shown in Fig. 18(A).

We thus conclude from Fig. 18 that expressing more than one gene at a stable steady state is only possible in a system with weak repression, whereas repression has essentially no effect on expression of genes not targeted by effector ${\bar{R}}_{2}$ and ${\bar{R}}_{3}$ here). Additionally, while weakening activation strength always expands the range of effector concentrations at which these stable fixed points can exist, only states that do not express the effector-targeted genes can exist at all effector concentrations (the yellow regions of Fig. 18). These regions require weaker activation strength compared to the strength of unregulated expression, with this requirement even more strict for the state expressing more than one gene. The results demonstrate that by tuning these parameters and testing varying effector concentrations, one can transform a system to express different numbers of genes at stable steady state depending on which combinations are most relevant for a desired function.

#### 2. Induction via Case (B)

An alternative interpretation for effector activity, shown in Case (B) of Fig. 11, transforms the available states and weights from the non-exclusive case such that the concentration of transcription factor ${\bar{R}}_{1}$ is scaled by $p_{\text{act}}(c)$ when repressing expression of other proteins, and scaled by $1-p_{\text{act}}(c)$ when activating its own expression (see Appendix B and corresponding Fig. 23 for the thermodynamic states and weights and for the corresponding model form).

**FIG. 19:**
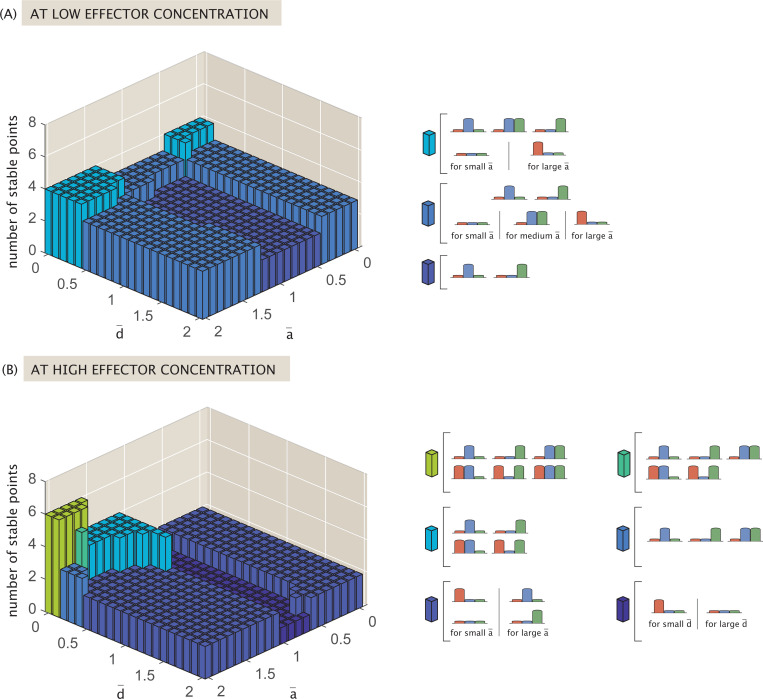
Number of stable fixed points in case (B) of non-exclusive repressor-activator binding. The plots highlight stable points at low (10^−8^ M) and high (10^−2^ M) effector concentrations in panels (A) and (B), respectively, for varying rate parameters $\bar{a}$, $\bar{b}$, and $\bar{d}$, where $\bar{a}+\bar{b}=2$ and $\bar{d}\leq \bar{a}+\bar{b}$. The diagrams on the right hand side of panel (A) represent the types of stable points observed in each regime at low effector concentration, i.e., with four (blue), three (dark blue), or two (purple) stable points. The diagrams on the right side of panel (B) represent the types of stable points observed in each regime at high effector concentration, i.e., with six (green), five (teal), four (blue), three (dark blue), two (purple), or one (dark purple) stable point(s).

By evaluating the resulting differential equations for fixed points, we obtain plots in Fig. 19 highlighting the types of stable points possible at low (10^−8^ M) and high (10^−2^ M) effector concentrations when tuning $\bar{a}$ and $\bar{d}$. Fig. 19(A) indicates that, regardless of activation strength $\left(\bar{a}\right)$ and repression $\left(\bar{d}\right)$, the system can always stabilize to a state in which either ${\bar{R}}_{2}$ or ${\bar{R}}_{3}$ dominates at low effector concentrations. When $0.5\leq \bar{a}≲1.3$ and $\bar{d}≳0.3$ (purple region), the system can only exist as a bistable switch between these two states. When repression is weak (small $\bar{d}$), the system can also stabilize to a state expressing both genes (regardless of $\bar{a}$). Meanwhile, the strength of activation solely determines whether it is possible for the system to suppress expression altogether or to stabilize to an ${\bar{R}}_{1}$-dominant state. For sufficiently strong activation $\left(\bar{a}<0.5\right)$, it is possible for regulation to suppress all expression, and it is only under weak activation $\left(\bar{a}≳1.3\right)$ that it is possible for the system to express the effector-targeted ${\bar{R}}_{1}$.

At high effector concentration, Fig. 19(B) indicates that the most complex dynamic landscape occurs when $\bar{d}$ is very small and $\bar{a}$ is very large, i.e., when there is effectively no positive or negative regulation in the network. Such a system can stabilize to either ${\bar{R}}_{2}$ or ${\bar{R}}_{3}$-dominant states, or to a state in which any combination of two or more proteins dominates (including effector-targeted ${\bar{R}}_{1}$), as shown in the green region. Moving slightly away from this corner, the system loses the ability to express all three genes, a change that is more sensitive to increased repression than increased activation. Increasing repression $\left(\bar{d}\right)$ while keeping $\bar{a}$ high suppresses the system’s ability to express effector-targeted ${\bar{R}}_{1}$. In this case, the dynamics become comparable to a self-activated bistable switch, as in the dark blue region of Fig. 19(B), or the simple bistable switch between ${\bar{R}}_{2}$ and ${\bar{R}}_{3}$, as in the purple region. Increasing activation (decreasing $\bar{a}$) while keeping repression relatively weak $\left(\bar{d}≲0.5\right)$, on the other hand, means that if the system expresses more than one gene in its stable state, one of them must be the effector-targeted ${\bar{R}}_{1}$, as seen in the blue region. In a strongly activating regime with small $\bar{a}$, dynamics center on expression of ${\bar{R}}_{1}$, either stabilizing to an ${\bar{R}}_{1}$-dominant state or suppressing all expression (purple region). If $\bar{a}\to 1$ as in the dark purple region of Fig. 19(B), however, the system chooses one of these fates depending on $\bar{d}$, only expressing a gene if repression ($\bar{d}$) remains small.

**FIG. 20:**
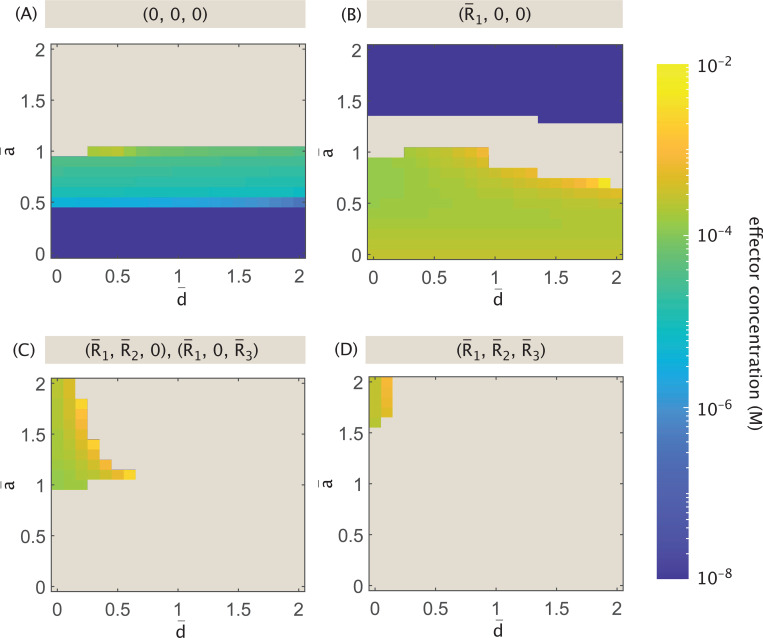
Heatmap tracking the minimum effector concentration (M) at which stable states can exist with non-exclusive activator-repressor binding model described in Fig. 11(B). Measurements span the rate parameters allowed by the constraints $\bar{a}+\bar{b}=2$ and $\bar{d}\leq \bar{a}+\bar{b}$, with the x-axis denoting increasing strength of repression $\bar{d}$, and the y-axis denoting decreasing strength of activation with increasing $\bar{a}$. The heatmaps track (A) the stable state in which no genes are expressed, (B) the stable state in which only the effector-targeted ${\bar{R}}_{1}$ is expressed, (C) stable states expressing two genes including the effector-controlled ${\bar{R}}_{1}$, and (D) the stable state expressing all three genes. Beige represents the part of parameter space in which the stable state cannot exist at any effector concentration.

**FIG. 21:**
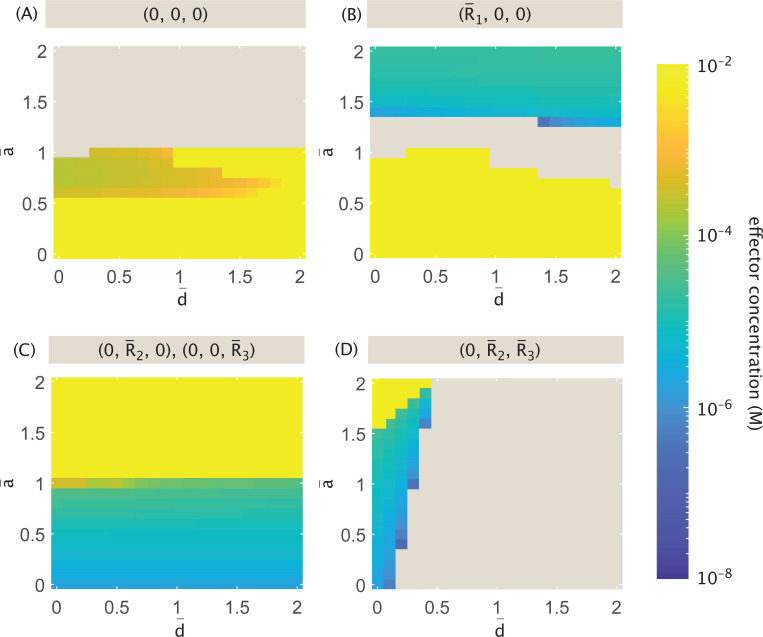
Heatmaps tracking the maximum effector concentrations (M) at which stable states can exist with non-exclusive activator-repressor binding model described in Fig. 11(B). The measured values represent bifurcation thresholds for the existence of these states. The heatmaps track (A) the stable state in which no genes are expressed, (B) the stable state in which only the effector-targeted ${\bar{R}}_{1}$ is expressed, (C) stable states expressing one of the genes that does not directly interact with effector, i.e., $\left(0,{\bar{R}}_{2},0\right)$ and $\left(0,0,{\bar{R}}_{3}\right)$, and (D) the stable state in which both genes not impacted by effector are expressed, i.e., $\left(0,{\bar{R}}_{2},{\bar{R}}_{3}\right)$. Measurements span the rate parameters allowed by the constraints $\bar{a}+\bar{b}=2$ and $\bar{d}\leq \bar{a}+\bar{b}$, with the x-axis denoting increasing strength of repression $\bar{d}$, and the y-axis denoting decreasing strength of activation as $\bar{a}$ rises. Beige represents the region where a stable state never exists at any effector concentration, and yellow indicates that the stable state is possible at all effector concentrations.

**FIG. 22:**
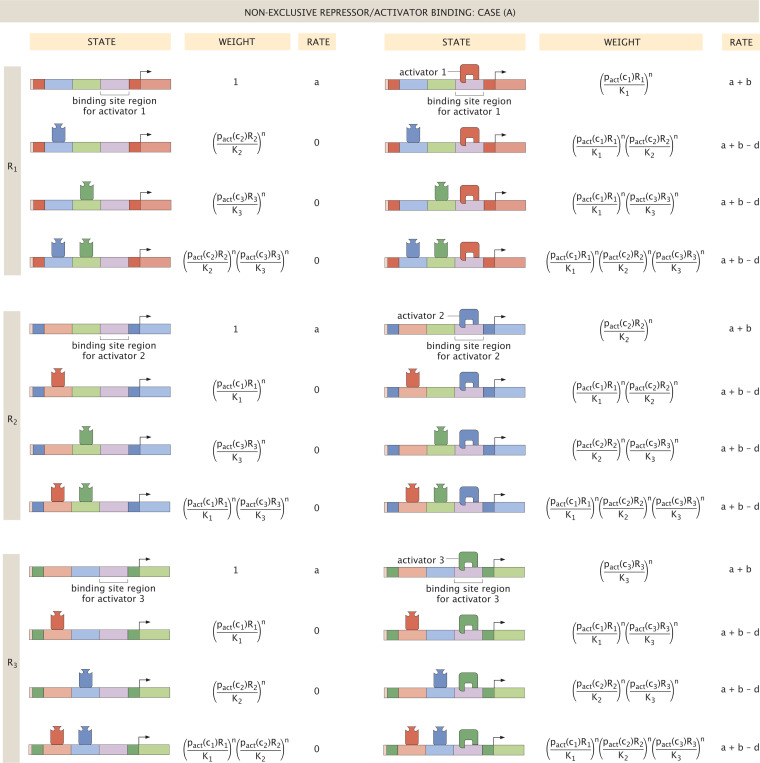
Expression of each protein $R_{i}$ in the three-gene toggle switch with self-activation and non-exclusive binding for repressors and activators. Effector binding is defined as in Fig. 11(A).

**FIG. 23:**
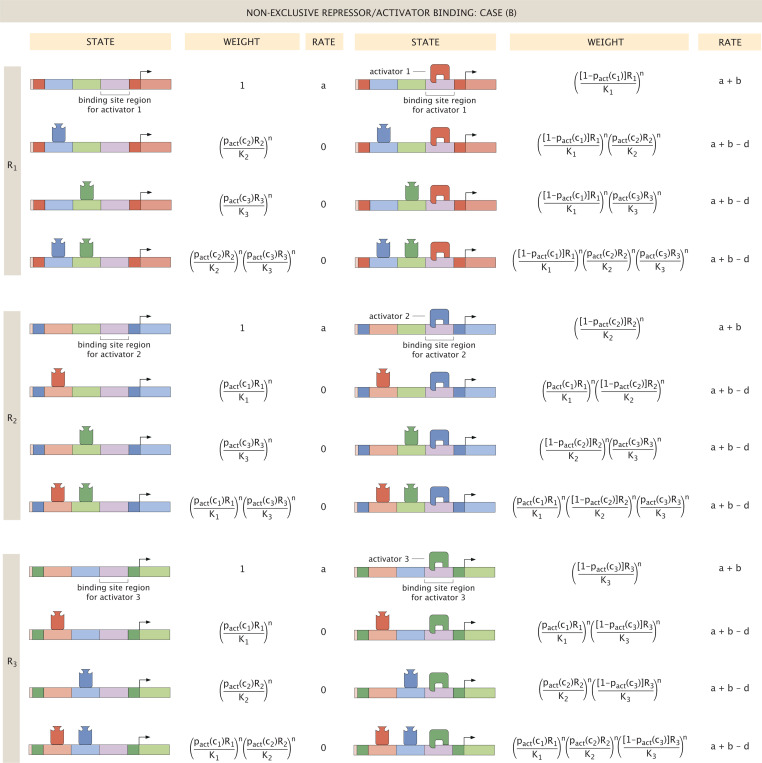
Expression of each protein $R_{i}$ in the three-gene toggle switch with self-activation and non-exclusive binding for repressors and activators. Effector binding is defined as in Fig. 11(B).

As previously shown in Fig. 17 and Fig. 18 for Case (A), we can also more closely investigate for Case (B) how different parametrizations for activation and repression influence the range of effector concentrations for which the different types of stable fixed points emerge. Fig. 20 tracks the minimum effector concentrations at which fixed points become possible within the parameter space, while Fig. 21 tracks the maximum effector concentrations. Given that Case (B) differs from Case (A) only in its treatment of ${\bar{R}}_{1}$, it follows that Fig. 18(A)-(B) and Fig. 21(C)-(D) show no difference in the expression patterns for those stable states that exclusively express one or both of the non-targeted genes, i.e., $\left(0,{\bar{R}}_{2},0\right)$, $\left(0,0,{\bar{R}}_{3}\right)$, and $\left(0,{\bar{R}}_{2},{\bar{R}}_{3}\right)$. A slight difference occurs for states expressing one of the two non-targeted genes when $\bar{a}=1$ and $\bar{d}\leq 0.5$. Under these conditions, where Case (A) would allow these states to exist at all effector concentrations, there is instead still an upper bound shown in Fig. 21(C) for Case (B) beyond which the states cannot exist. This means that when effectors determine whether a protein acts a repressor or an activator, and the system neither favors nor disfavors activation, minimizing the effect of repression is not sufficient to allow expression at all effector concentrations, and that this can only be true if the system explicitly favors activation with $\bar{a}>1$.

States expressing the effector-targeted ${\bar{R}}_{1}$ in Case (B) offer a stark contrast to those in Case (A). In Case (A), the ${\bar{R}}_{1}$-dominant stable state was not included amongst the plots of Fig. 17 and Fig. 18 because it can exist for essentially the same range of low effector concentrations (from 10^−8^ M to approximately 5×10^−5^ M) regardless of $\bar{a}$ and $\bar{d}$. In Case (B), however, this is only observed for $\bar{a}≳1.4$, as seen in Fig. 20(B) and Fig. 21(B). Further, if $\bar{a}\leq 1$ (with some exceptions), the state can only exist at larger effector concentrations ranging from approximately 10^−4^ M to the highest sampled 10^−2^ M. Thus, when activation is particularly strong, the phase space for ${\bar{R}}_{1}$-dominant expression spans the same range of concentrations observed for Case (A). When activation is disfavored as the source of protein production compared to the basal level of expression, however, ${\bar{R}}_{1}$-dominant expression emerges as a viable stable state only at high effector concentrations lying outside of the previously established range.

Considering the stable state in which all expression is suppressed, Fig. 17 and Fig. 20(A) demonstrate that regardless of the effector’s role in regulating ${\bar{R}}_{1}$, this type of stable state can exist only when activation is favored $\left(\bar{a}\leq 1\right)$ over the basal rate. The strong resemblance between these plots also indicates that, in the viable region of parameter space, tuning $\bar{a}$ and $\bar{d}$ similarly affects the minimum concentration necessary for the state to exist. We observe small differences at the two visible thresholds: $\bar{a}=0.5$ separates the high-activation regime in which the state always exists at low effector concentration from the regime characterized by a particular threshold effector concentration; and $\bar{a}=1$ denotes the upper bound of $\bar{a}$ for which the (0,0,0) state can exist at any effector concentration. Compared to Case (A), when $\bar{a}=1$ Case (B) requires an even weaker level of repression $\bar{d}$ to prevent the system from being able to suppress all expression.

Unlike Case (A), for Case (B) the existence of the (0,0,0) stable state does not immediately imply its viability for all effector concentrations above the minimum threshold. Fig. 21(A) shows that, in a regime of intermediate activation strength where $0.6\leq \bar{a}\leq 1$, there is an upper bound on the effector concentration. These intermediate conditions mark a regime in which the effector concentration can be manipulated to tune stable expression off and on.

Finally, we consider stable states expressing more than one protein including the effector-targeted ${\bar{R}}_{1}$. Whereas Fig. 18(C)-(D) indicates that these states can exist for Case (A) at low effector concentrations up to values between 10^−6^ M and 10^−5^ M when repression $\bar{d}$ is small, Fig. 20(C)-(D) indicates an overlapping regime of existence but with distinctly different dependencies on both rate parameters and on effector concentrations. In fact, these panels reveal that such states can only exist at higher effector concentrations above approximately 10^−4^ M, and that they further rely on there being sufficiently weak activation.

## VI. DISCUSSION

Significant strides have been made since the foundational work of the 1960s to uncover the mechanisms by which cells regulate gene expression and the interactions within vast gene networks that facilitate nuanced changes in expression. This has led to a large body of both experimental and theoretical work studying a number of commonly observed motifs including simple two-gene switches, oscillators, and feed-forward networks, among others [1, 14, 16, 22, 78–84]. Even with these significant advances in knowledge, more remains to be explored. In fact, for *E. coli*, perhaps the most well-studied simple model organism, we still do not understand how more than ∼ 60% of its genes are regulated [85]. As the field progresses in obtaining and interpreting high-throughput data, we will likely uncover additional aspects of gene regulation that require more nuanced modeling. We expand upon the valuable existing work to consider a range of other motifs for modeling gene regulatory expression.

Much existing literature from a statistical physics perspective (and outside the realm of machine learning in high-dimensional networks) focuses on networks that ultimately tune expression of a single protein on or off, and rely on small-scale motifs. Our primary interest lies in biological settings with many cell fates, particularly in developmental and immune processes, that clearly rely on coordinated regulatory efforts in the expression of multiple genes. This type of analysis calls for consideration of higher-order gene regulatory motifs such as the three-gene toggle switch we implement.

There is also still much to be learned about the proteins responsible for gene regulation. In particular, we do not fully understand the mechanisms by which effector molecules control the transcription factors that regulate expression. Given the prevalence of induction in the biophysical literature as an experimental tool for tuning expression, we are further motivated to use the three-gene toggle switch to consider allosteric regulation. Importantly, by studying an intricate network that can involve both repression and activation, we can also explore how the biological interpretation of allosteric induction impacts dynamics.

At the core of our analysis is a baseline model of three genes in which each gene represses all others. By harnessing inducers as tuning knobs controlling the concentration of active repressor(s), we observe how tuning activity through an MWC interpretation of allosteric induction limits or broadens the scope of dynamics, and how robust (or sensitive) bifurcations separating different dynamic regimes are to changes in cooperativity and relative gene interaction strengths.

In the first half of the paper we observe that the simple three-gene toggle switch, in which only one of the three genes can dominate expression in a stable state, follows a trajectory of decreasing dynamic complexity as inducer concentration(s) increase. There are two notable takeaways from analysis of the three dynamic regimes that become possible. First, we observe that the bifurcation separating bistable from tristable dynamics varies little as the strength of cooperativity increases, and that the maximum level of gene expression observed remains consistent as inducer concentrations rise. This facilitates the fitting of model parameters to experimental data, since altering the $K_{I}$ of our $p_{\text{act}}(c)$ function becomes the only way to noticeably shift this bifurcation threshold. Meanwhile, when not at a low cooperativity level, the bifurcation separating different tristable regimes depends most strongly on perturbation of the model’s regulatory interaction structure away from symmetry. By determining the probabilities of stabilizing at each of the possible three stable points from different initial conditions at a low inducer concentration, one can determine how close the system is to the “symmetric” setting (i.e., equally strong repression among all genes) that we model. If the system tuned by one inducer is instead bistable at all inducer concentrations, it follows that this arises from a strongly skewed set of repression strengths explicitly favoring one gene, rather than from allosteric regulation.

While the baseline model demonstrates the utility of allosteric regulation as a tool for identifying the physical parameters driving expression in experimental data, the second half of the paper highlights how the mechanism by which allosteric regulation controls transcription factor activity can significantly alter the dynamics. We show this in a model for the three-gene toggle switch that allows for self-activation. Introducing activators that competitively bind to sites along the DNA does not affect the stable expression levels observed in the baseline model, but decreases the probability of transitioning from one stable state to another (without increased noise), placing stable states in deeper potential wells within the potential landscape. When self-activation is sufficiently strong, or when activators and repressors bind non-exclusively along the DNA, it becomes possible for the system to stabilize while expressing more than one gene. Understanding the parameter conditions, including the ranges of effector concentrations, that allow different stable states to emerge is a useful tool for determining how cells tune activity to coordinate expression of multiple genes.

We also find across several case studies with competitive and non-exclusive repressor/activator binding that the biological interpretation of the effector’s role in regulation matters, whether an effector’s binding determines activity regardless of function as a repressor or an activator, or whether it directly determines function. Considering first systems in which activators compete with repressors to bind along the DNA, changing the effector’s role does not alter either the types of dynamic phases possible or the responses to increasing effector concentrations. In the case where the effector determines protein function, however, the effector concentrations at which bifurcations occur shift to higher values, and it becomes easier to transition out of an ${\bar{R}}_{1}$-dominant state and more difficult to transition out of an ${\bar{R}}_{2}$ or ${\bar{R}}_{3}$ dominant state. In the non-exclusive binding settings, our findings under the two interpretations of the effector reveal distinct differences in the types of phases observed across parameter space, as well as in the ranges of effector concentrations at which different types of stable points could be observed.

Tuning effector concentration(s) thus allows us to distinguish among these biologically distinct models for the same gene regulatory motif, even when the particular set of stable states observed is characteristic to multiple models. For instance, in both the competitive (with strong activation) and non-exclusive binding settings, it is possible for the system to stabilize at low effector concentrations to any of the three single-gene-dominant states, or to a state suppressing all expression. As the effector concentration increases, however, the candidate models have divergent responses and are thus distinguishable. If the system becomes tristable to either suppress all expression or allow expression of one of the genes not targeted by the effector, the system identifies as competitive repressor-activator binding. If expression becomes suppressed entirely, the system instead allows non-exclusive binding, with the binding site determining transcription factor function (and the effector only determining protein activity). Finally, if the system transforms to a bistable switch between no expression and expression of the effector-targeted gene alone, this implies non-exclusive binding where effector presence directly determines transcription factor function.

The mathematical interpretation of the biological mechanisms for induction embedded in the models we study have a significant impact on the types of dynamics observed within parameter space. Our analysis therefore complements existing work on the direct incorporation of effector-driven transcription factor activity into models of gene regulation while further motivating the rigorous definition of *how* effectors function. In so doing, the work presents a theoretical approach through which it would be feasible to uncover such properties (and the model parameters that allow them) in conjunction with experiments. As evidenced by the diverse outputs that are possible from the three-noded networks studied here when subjected to a range of effector inputs, there is considerable richness to the input-output responses of these systems. Our findings underscore the importance and the challenge of understanding how such outcomes are realized in even more complex regulatory architectures, and invite dialogue with ongoing experimental efforts to reveal the full scope of the allosterome.

## Appendix A: Analytic bifurcations for the two inducer model

In this appendix, we show analytically that the linear thresholds separating $\left(c_{1},c_{2}\right)$ parameter space into tristable, bistable, and monostable regions in Section IIIB are the same as those derived for the single inducer model in Section IIIA.

The first key bifurcation indicator with respect to $c_{1}$ is the loss of the ${\bar{R}}_{1}$-dominant stable state in transitioning from tristable to bistable dynamics. Assuming that ${\bar{R}}_{2,3}^{n}\to 0$ as in the single inducer setting, Eqns. 31 – 33 indicate a steady state solution

(A1)
$$\begin{array}{l} {\bar{R}}_{ss}=\left({\bar{R}}_{1},{\bar{R}}_{2},{\bar{R}}_{3}\right)\\ \;=\left(\bar{a},\frac{\bar{a}}{1+{\left[p_{\text{act}}\left(c_{1}\right)\bar{a}\right]}^{n}},\frac{\bar{a}}{1+{\left[p_{\text{act}}\left(c_{1}\right)\bar{a}\right]}^{n}}\right). \end{array}$$

Since our assumption of the relative expression levels requires ${\bar{R}}_{2}={\bar{R}}_{3}\leq \varepsilon$, it follows that

(A2)
$$\varepsilon \geq \frac{\bar{a}}{1+{\left[p_{\text{act}}\left(c_{1}\right)\bar{a}\right]}^{n}}$$

and thus that the ${\bar{R}}_{1}$ dominant steady state exists for

(A3)
$$p_{\text{act}}\left(c_{1}\right)\geq \frac{1}{\bar{a}}\sqrt[{n}]{\frac{\bar{a}}{\varepsilon }-1},$$

which is the same as the result derived in Eqn. 29 in the case of the single inducer.

We can derive the same result for $p_{\text{act}}\left(c_{2}\right)$ by determining the bifurcation point with respect to $c_{2}$ at which the system no longer stabilizes to an ${\bar{R}}_{2}$-dominant state.

## Appendix B: Non-exclusive repressor-activator binding in the three-gene toggle switch

In Section VB we discuss the three-gene toggle switch with self-activation where repressors and activators can bind non-exclusively at a gene’s regulatory region. This appendix outlines the analytic model and how it is modified under different interpretations of effector activity.

Fig. 22 shows the thermodynamic states, weights and rates related to non-exclusive binding. Note that these include the same states (and corresponding rates) describing competitive binding in Fig. 12(A), but with three additional states in which an activator and either one or two repressors can be bound simultaneously at the relevant gene regulatory site. In these additional states, the presence of repressor(s) dampens expression from its activated rate to a+b−d. The states and weights depicted translate into a corresponding system of equations for the evolution of expression. Omitting the role of effector, the change in $R_{1}$ expression, for instance, is

(B1)
$$\begin{array}{l} \frac{dR_{1}}{dt}=\frac{a+(a+b){\left(\frac{R_{1}}{K_{1}}\right)}^{n}+(a+b-d){\left(\frac{R_{1}}{K_{1}}\right)}^{n}\left[{\left(\frac{R_{2}}{K_{2}}\right)}^{n}+{\left(\frac{R_{3}}{K_{3}}\right)}^{n}+{\left(\frac{R_{2}}{K_{2}}\right)}^{n}{\left(\frac{R_{3}}{K_{3}}\right)}^{n}\right]}{\left[1+{\left(\frac{R_{1}}{K_{1}}\right)}^{n}\right]\left[1+{\left(\frac{R_{2}}{K_{2}}\right)}^{n}\right]\left[1+{\left(\frac{R_{3}}{K_{3}}\right)}^{n}\right]}-\frac{R_{1}}{\tau }\\ \;=\frac{a}{\left[1+{\left(\frac{R_{2}}{K_{2}}\right)}^{n}\right]\left[1+{\left(\frac{R_{3}}{K_{3}}\right)}^{n}\right]}+\frac{b{\left(\frac{R_{1}}{K_{1}}\right)}^{n}}{1+{\left(\frac{R_{1}}{K_{1}}\right)}^{n}}+\frac{(a-d){\left(\frac{R_{1}}{K_{1}}\right)}^{n}\left[{\left(\frac{R_{2}}{K_{2}}\right)}^{n}+{\left(\frac{R_{3}}{K_{3}}\right)}^{n}+{\left(\frac{R_{2}}{K_{2}}\right)}^{n}{\left(\frac{R_{3}}{K_{3}}\right)}^{n}\right]}{\left[1+{\left(\frac{R_{1}}{K_{1}}\right)}^{n}\right]\left[1+{\left(\frac{R_{2}}{K_{2}}\right)}^{n}\right]\left[1+{\left(\frac{R_{3}}{K_{3}}\right)}^{n}\right]}-\frac{R_{1}}{\tau }, \end{array}$$

where a is the rate of protein production when gene transcription is unregulated, a+b is the rate with bound self-activator, and a+b−d is the previous rate attenuated by the presence of bound repressor. Note that the second line of Eqn. B1 rearranges the first to separate protein production into three physically meaningful terms: (i) protein expression in the presence of repressors only, (ii) expression exclusively due to activation, and (iii) expression in the presence of both repressors and activators. Rescaling variables in Eq. B1 and in the equivalent equations for $R_{2}$ and $R_{3}$ expression such that ${\bar{R}}_{i}=R_{i}/K_{1}$, $\bar{t}=t/\tau$, $\bar{a}=\tau a/K_{1}$, $\bar{b}=\tau b/K_{i}$, and $\bar{d}=\tau d/K_{1}$, we define our non-exclusive

binding model in dimensionless form by the set of differential equations

(B2)
$$\frac{d{\bar{R}}_{1}}{d\bar{t}}=\frac{\bar{a}}{\left[1+{\left(\frac{{\bar{R}}_{2}}{K^{\left(2\right)}}\right)}^{n}\right]\left[1+{\left(\frac{{\bar{R}}_{3}}{K^{\left(3\right)}}\right)}^{n}\right]}+\frac{\bar{b}{\bar{R}}_{1}^{n}}{1+{\bar{R}}_{1}^{n}}+\frac{(\bar{a}-\bar{d}){\bar{R}}_{1}^{n}\left[{\left(\frac{{\bar{R}}_{2}}{K^{\left(2\right)}}\right)}^{n}+{\left(\frac{{\bar{R}}_{3}}{K^{\left(3\right)}}\right)}^{n}+{\left(\frac{{\bar{R}}_{2}}{K^{\left(2\right)}}\right)}^{n}{\left(\frac{{\bar{R}}_{3}}{K^{\left(3\right)}}\right)}^{n}\right]}{\left[1+{\bar{R}}_{1}^{n}\right]\left[1+{\left(\frac{{\bar{R}}_{2}}{K^{\left(2\right)}}\right)}^{n}\right]\left[1+{\left(\frac{{\bar{R}}_{3}}{K^{\left(3\right)}}\right)}^{n}\right]}-{\bar{R}}_{1},$$

(B3)
$$\frac{d{\bar{R}}_{2}}{d\bar{t}}=\frac{\bar{a}}{\left[1+{\bar{R}}_{1}^{n}\right]\left[1+{\left(\frac{{\bar{R}}_{3}}{K^{\left(3\right)}}\right)}^{n}\right]}+\frac{\bar{b}{\left(\frac{{\bar{R}}_{2}}{K^{\left(2\right)}}\right)}^{n}}{1+{\left(\frac{{\bar{R}}_{2}}{K^{\left(2\right)}}\right)}^{n}}+\frac{(\bar{a}-\bar{d}){\left(\frac{{\bar{R}}_{2}}{K^{\left(2\right)}}\right)}^{n}\left({\bar{R}}_{1}^{n}+{\left(\frac{{\bar{R}}_{3}}{K^{\left(3\right)}}\right)}^{n}+{\bar{R}}_{1}^{n}{\left(\frac{{\bar{R}}_{3}}{K^{\left(3\right)}}\right)}^{n}\right)}{\left[1+{\bar{R}}_{1}^{n}\right]\left[1+{\left(\frac{{\bar{R}}_{2}}{K^{\left(2\right)}}\right)}^{n}\right]\left[1+{\left(\frac{{\bar{R}}_{3}}{K^{\left(3\right)}}\right)}^{n}\right]}-{\bar{R}}_{2},$$

(B4)
$$\frac{d{\bar{R}}_{3}}{d\bar{t}}=\frac{\bar{a}}{\left[1+{\bar{R}}_{1}^{n}\right]\left[1+{\left(\frac{{\bar{R}}_{2}}{K^{\left(2\right)}}\right)}^{n}\right]}+\frac{\bar{b}{\left(\frac{{\bar{R}}_{3}}{K^{\left(3\right)}}\right)}^{n}}{1+{\left(\frac{{\bar{R}}_{3}}{K^{\left(3\right)}}\right)}^{n}}+\frac{(\bar{a}-\bar{d}){\left(\frac{{\bar{R}}_{3}}{K^{\left(3\right)}}\right)}^{n}\left({\bar{R}}_{1}^{n}+{\left(\frac{{\bar{R}}_{2}}{K^{\left(2\right)}}\right)}^{n}+{\bar{R}}_{1}^{n}{\left(\frac{{\bar{R}}_{2}}{K^{\left(2\right)}}\right)}^{n}\right)}{\left[1+{\bar{R}}_{1}^{n}\right]\left[1+{\left(\frac{{\bar{R}}_{2}}{K^{\left(2\right)}}\right)}^{n}\right]\left[1+{\left(\frac{{\bar{R}}_{3}}{K^{\left(3\right)}}\right)}^{n}\right]}-{\bar{R}}_{3},$$

with $\bar{d}\leq \bar{a}+\bar{b}$, $K^{\left(2\right)}=K_{2}/K_{1}$, and $K^{\left(3\right)}=K_{3}/K_{1}$. As in Section VA1, our analysis for this model assumes equal binding affinities such that $K^{\left(2\right)}=K^{\left(3\right)}=1$, and assumes a maximal production rate $\bar{a}+\bar{b}=2$.

Unlike the competitive binding case, the model defined in Eqns. B2 - B4 depends on *two* independent parameters: (i) unregulated (basal) protein expression $\bar{a}$, with corresponding $\bar{b}=2-\bar{a}$ denoting additional expression from activations, and (ii) the strength of repression from $\bar{d}$. We now explicitly incorporate the role of effector molecules in two ways.

First, Fig. 22 defines effector activity as described by Case (A) of Fig. 11, where the active concentration of a protein indexed by i is $p_{\text{act}}(c){\bar{R}}_{i}$ regardless of its function as a repressor or an activator. Therefore, for the system containing one effector at concentration c that targets ${\bar{R}}_{1}$, the dimensionless Eqns. B2 - B4 in induction Case (A) become

(B5)
$$\frac{d{\bar{R}}_{1}}{d\bar{t}}=\frac{\bar{a}}{\left[1+{\bar{R}}_{2}^{n}\right]\left[1+{\bar{R}}_{3}^{n}\right]}+\frac{\bar{b}{\left[p_{\text{act}}(c){\bar{R}}_{1}\right]}^{n}}{1+{\left[p_{\text{act}}(c){\bar{R}}_{1}\right]}^{n}}+\frac{(\bar{a}-\bar{d}){\left[p_{\text{act}}(c){\bar{R}}_{1}\right]}^{n}\left[{\bar{R}}_{2}^{n}+{\bar{R}}_{3}^{n}+{\bar{R}}_{2}^{n}{\bar{R}}_{3}^{n}\right]}{\left[1+{\left[p_{\text{act}}(c){\bar{R}}_{1}\right]}^{n}\right]\left[1+{\bar{R}}_{2}^{n}\right]\left[1+{\bar{R}}_{3}^{n}\right]}-{\bar{R}}_{1},$$

(B6)
$$\frac{d{\bar{R}}_{2}}{d\bar{t}}=\frac{\bar{a}}{\left[1+{\left[p_{\text{act}}(c){\bar{R}}_{1}\right]}^{n}\right]\left[1+{\bar{R}}_{3}^{n}\right]}+\frac{\bar{b}{\bar{R}}_{2}^{n}}{1+{\bar{R}}_{2}^{n}}+\frac{(\bar{a}-\bar{d}){\bar{R}}_{2}^{n}\left({\left[p_{\text{act}}(c){\bar{R}}_{1}\right]}^{n}+{\bar{R}}_{3}^{n}+{\left[p_{\text{act}}(c){\bar{R}}_{1}\right]}^{n}{\bar{R}}_{3}^{n}\right)}{\left[1+{\left[p_{\text{act}}(c){\bar{R}}_{1}\right]}^{n}\right]\left[1+{\bar{R}}_{2}^{n}\right]\left[1+{\bar{R}}_{3}^{n}\right]}-{\bar{R}}_{2},$$

(B7)
$$\frac{d{\bar{R}}_{3}}{d\bar{t}}=\frac{\bar{a}}{\left[1+{\left[p_{\text{act}}(c){\bar{R}}_{1}\right]}^{n}\right]\left[1+{\bar{R}}_{2}^{n}\right]}+\frac{\bar{b}{\bar{R}}_{3}^{n}}{1+{\bar{R}}_{3}^{n}}+\frac{(\bar{a}-\bar{d}){\bar{R}}_{3}^{n}\left({\left[p_{\text{act}}(c){\bar{R}}_{1}\right]}^{n}+{\bar{R}}_{2}^{n}+{\left[p_{\text{act}}(c){\bar{R}}_{1}\right]}^{n}{\bar{R}}_{2}^{n}\right)}{\left[1+{\left[p_{\text{act}}(c){\bar{R}}_{1}\right]}^{n}\right]\left[1+{\bar{R}}_{2}^{n}\right]\left[1+{\bar{R}}_{3}^{n}\right]}-{\bar{R}}_{3}.$$

Evaluating Eqns. B5 - B7 for fixed points, we obtain the results discussed in Section VB1.

An alternative interpretation for effector activity, described by Case (B) of Fig. 11, transforms the available states and weights to those in Fig. 23, where the concentration of ${\bar{R}}_{i}$ is scaled by $p_{\text{act}}(c)$ when repressing expression of other proteins, and scaled by $1-p_{\text{act}}(c)$ when activating its own expression. For the system containing only one effector at concentration c targetting ${\bar{R}}_{1}$ activity, Eqns. B2 - B4 thus become

(B8)
$$\frac{d{\bar{R}}_{1}}{d\bar{t}}=\frac{\bar{a}}{\left[1+{\bar{R}}_{2}^{n}\right]\left[1+{\bar{R}}_{3}^{n}\right]}+\frac{\bar{b}{\left[\left(1-p_{\text{act}}(c)\right){\bar{R}}_{1}\right]}^{n}}{1+{\left[\left(1-p_{\text{act}}(c)\right){\bar{R}}_{1}\right]}^{n}}+\frac{(\bar{a}-\bar{d}){\left[\left(1-p_{\text{act}}(c)\right){\bar{R}}_{1}\right]}^{n}\left[{\bar{R}}_{2}^{n}+{\bar{R}}_{3}^{n}+{\bar{R}}_{2}^{n}{\bar{R}}_{3}^{n}\right]}{\left[1+{\left[\left(1-p_{\text{act}}(c)\right){\bar{R}}_{1}\right]}^{n}\right]\left[1+{\bar{R}}_{2}^{n}\right]\left[1+{\bar{R}}_{3}^{n}\right]}-{\bar{R}}_{1},$$

(B9)
$$\frac{d{\bar{R}}_{2}}{d\bar{t}}=\frac{\bar{a}}{\left[1+{\left[p_{\text{act}}(c){\bar{R}}_{1}\right]}^{n}\right]\left[1+{\bar{R}}_{3}^{n}\right]}+\frac{\bar{b}{\bar{R}}_{2}^{n}}{1+{\bar{R}}_{2}^{n}}+\frac{(\bar{a}-\bar{d}){\bar{R}}_{2}^{n}\left({\left[p_{\text{act}}(c){\bar{R}}_{1}\right]}^{n}+{\bar{R}}_{3}^{n}+{\left[p_{\text{act}}(c){\bar{R}}_{1}\right]}^{n}{\bar{R}}_{3}^{n}\right)}{\left[1+{\left[p_{\text{act}}(c){\bar{R}}_{1}\right]}^{n}\right]\left[1+{\bar{R}}_{2}^{n}\right]\left[1+{\bar{R}}_{3}^{n}\right]}-{\bar{R}}_{2},$$

(B10)
$$\frac{d{\bar{R}}_{3}}{d\bar{t}}=\frac{\bar{a}}{\left[1+{\left[p_{\text{act}}(c){\bar{R}}_{1}\right]}^{n}\right]\left[1+{\bar{R}}_{2}^{n}\right]}+\frac{\bar{b}{\bar{R}}_{3}^{n}}{1+{\bar{R}}_{3}^{n}}+\frac{(\bar{a}-\bar{d}){\bar{R}}_{3}^{n}\left({\left[p_{\text{act}}(c){\bar{R}}_{1}\right]}^{n}+{\bar{R}}_{2}^{n}+{\left[p_{\text{act}}(c){\bar{R}}_{1}\right]}^{n}{\bar{R}}_{2}^{n}\right)}{\left[1+{\left[p_{\text{act}}(c){\bar{R}}_{1}\right]}^{n}\right]\left[1+{\bar{R}}_{2}^{n}\right]\left[1+{\bar{R}}_{3}^{n}\right]}-{\bar{R}}_{3}.$$

Evaluating Eqns. B8 - B10 for fixed points, we obtain the results discussed in Section VB2.

## References

[1] Ozbudak E. M., Thattail M., Lim H. N., Shraiman B. I., and van Oudenaarden A., Multistability in the lactose utilization network of *Escherichia coli*, Nature 427, 737 (2004). 14973486 10.1038/nature02298

[2] Sheth R., Marcon L., Bastida M. F., Junco M., Quintana L., Dahn R., Kmita M., Sharpe J., and Ros M. A., *Hox* Genes Regulate Digit Patterning by Controlling the Wavelength of a Turing-Type Mechanism, Science 338, 1476 (2012). 23239739 10.1126/science.1226804 PMC4486416

[3] Raspopovic J., Marcon L., Russo L., and Sharpe J., Digit patterning is controlled by a Bmp-Sox9-Wnt Turing network modulated by morphogen gradients, Science 345, 566 (2014). 25082703 10.1126/science.1252960

[4] Zuniga A. and Zeller R., In Turing’s hands—the making of digits, Science 345, 516 (2014). 25082687 10.1126/science.1257501

[5] Sternberg P. W. and Horvitz H. R., The combined action of two intercellular signaling pathways specifies three cell fates during vulval induction in *C. elegans*, Cell 58, 679 (1989). 2548732 10.1016/0092-8674(89)90103-7

[6] Hoyos E., Kim K., Milloz J., Barkoulas M., Pénigault J. B., Munro E., and Félix M. A., Quantitative Variation in Autocrine Signaling and Pathway Crosstalk in the *Caenorhabditis* Vulval Network, Current Biology 21, 527 (2011). 21458263 10.1016/j.cub.2011.02.040 PMC3084603

[7] Takahashi K. and Yamanaka S., Induction of Pluripotent Stem Cells from Mouse Embryonic and Adult Fibroblast Cultures by Defined Factors, Cell 126, 663 (2006). 16904174 10.1016/j.cell.2006.07.024

[8] Hanna J. H., Saha K., and Jaenisch R., Pluripotency and Cellular Reprogramming: Facts, Hypotheses, Unresolved Issues, Cell 143, 508 (2010). 21074044 10.1016/j.cell.2010.10.008 PMC3032267

[9] Jia D., Jolly M. K., Kulkarni P., and Levine H., Phenotypic Plasticity and Cell Fate Decisions in Cancer: Insights from Dynamical Systems Theory, Cancers 9, 70 (2017). 28640191 10.3390/cancers9070070 PMC5532606

[10] Johnson A. D., Poteete A. R., Lauer G., Sauer R. T., Ackers G. K., and Ptashne M., *λ* Repressor and cro–components of an efficient molecular switch, Nature 294, 217 (1981). 6457992 10.1038/294217a0

[11] Ptashne M. and Gann A., Genes and Signals (Cold Spring Harbor Laboratory Press, Cold Spring Harbor, N.Y., 2002).

[12] Ptashne M., A Genetic Switch: Phage Lambda Revisited, 3rd ed. (Cold Spring Harbor Laboratory Press, Cold Spring Harbor, N.Y., 2004).

[13] Golding I., Decision Making in Living Cells: Lessons from a Simple System, Annu. Rev. Biophys. 40, 63 (2011). 10.1146/annurev-biophys-042910-155227 PMC4465489 21545284

[14] Gardner T. S., Cantor C. R., and Collins J. J., Construction of a genetic toggle switch in *Escherichia coli*, Nature 403, 339 (2000). 10659857 10.1038/35002131

[15] Cherry J. L. and Adler F. R., How to make a biological switch, J. Theor. Biol. 203, 117 (2000). 10704297 10.1006/jtbi.2000.1068

[16] Huang S., Guo Y. P., May G., and Enver T., Bifurcation dynamics in lineage-commitment in bipotent progenitor cells, Developmental Biology 305, 695 (2007). 17412320 10.1016/j.ydbio.2007.02.036

[17] Waddington C. H., The Strategy of the Genes (Routledge, 1957).

[18] Corson F. and Siggia E. D., Geometry, epistasis, and developmental patterning, Proc. Natl. Acad. Sci. U.S.A. 109, 5568 (2012). 22434912 10.1073/pnas.1201505109 PMC3326455

[19] Corson F. and Siggia E. D., Gene-free methodology for cell fate dynamics during development, eLife 6, e30743 (2017). 10.7554/eLife.30743 PMC5771671 29235987

[20] Rand D. A., Raju A., Sáez M., Corson F., and Siggia E. D., Geometry of gene regulatory dynamics, Proc. Natl. Acad. Sci. U.S.A. 118, e2109729118 (2021). 10.1073/pnas.2109729118 PMC8463785 34518231

[21] Sáez M., Briscoe J., and Rand D. A., Dynamical landscapes of cell fate decisions, Interface Focus 12, 20220002 (2022). 10.1098/rsfs.2022.0002 PMC9184965 35860004

[22] Phillips R., The Molecular Switch: Signaling and Allostery (Princeton University Press, 2020).

[23] Gerhart J. C. and Pardee A. B., Enzymology of control by feedback inhibition, J. Biol. Chem. 237, 891 (1962). 13897943

[24] Monod J., Changeux J. P., and Jacob F., Allosteric proteins and cellular control systems, J. Mol. Biol. 6, 306 (1963). 13936070 10.1016/s0022-2836(63)80091-1

[25] Monod J., Wyman J., and Changeux J. P., On the nature of allosteric transitions: a plausible model, J. Mol. Biol. 12, 88 (1965). 14343300 10.1016/s0022-2836(65)80285-6

[26] O’Gorman R. B., Rosenberg J. M., Kallai O. B., Dickerson R. E., Itakura K., Riggs A. D., and Matthews K. S., Equilibrium binding of inducer to *lac* repressor-operator DNA complex, J. Biol. Chem. 255, 10107 (1980). 7000771

[27] Daly T. J. and Matthew K. S., Characterization and modification of a monomeric mutant of the lactose repressor protein, Biochemistry 25, 5474 (1986). 3535879 10.1021/bi00367a019

[28] Martins B. M. and Swain P. S., Trade-offs and constraints in allosteric sensing, PLoS Comput. Biol. 7, e1002261 (2011). 10.1371/journal.pcbi.1002261 PMC3207937 22096453

[29] Marzen S., Garcia H. G., and Phillips R., Statistical mechanics of Monod-Wyman-Changeux (MWC) models, J. Mol. Biol. 425, 1433 (2013). 23499654 10.1016/j.jmb.2013.03.013 PMC3786005

[30] Changeux J. P., 50 years of allosteric interactions: the twists and turns of the models, Nat. Rev. Mol. Cell Biol. 14, 819 (2013). 24150612 10.1038/nrm3695

[31] Gerhart J., From feedback inhibition to allostery: the enduring example of aspartate transcarbamoylase, FEBS J. 281, 612 (2014). 23953008 10.1111/febs.12483

[32] Alberts B., Heald R., Johnson A., Morgan D., Raff M., Roberts K., and Walter P., Molecular Biology of the Cell (Garland Science, N.Y., 2015) Chap. 7, 6th ed.

[33] Genuario R. R. and Perry R. P., The GA-binding protein can serve as both an activator and repressor of ribosomal protein gene transcription, J. Biol. Chem. 271, 4388 (1996). 8626789 10.1074/jbc.271.8.4388

[34] Marbach A. and Bettenbrock K., lac operon induction in Escherichia coli: Systematic comparison of IPTG and TMG induction and influence of the transacetylase LacA, J. Biotech. 157, 82 (2012). 10.1016/j.jbiotec.2011.10.009 22079752

[35] Santillán M. and Mackey M. C., Influence of catabolite repression and inducer exclusion on the bistable behavior of the *lac* operon, Biophys. J. 86, 1282 (2004). 14990461 10.1016/S0006-3495(04)74202-2 PMC1303969

[36] Fang D. and Zhu J., Dynamic balance between master transcription factors determines the fates and functions of CD4 T cell and innate lymphoid cell subsets, J. Exp. Med. 214, 1861 (2010). 10.1084/jem.20170494 PMC5502437 28630089

[37] Swain N., Thakur M., Pathak J., and Swain B., SOX2, OCT4 and NANOG: The core embryonic stem cell pluripotency regulators in oral carcinogenesis, J. Oral Maxillofac. Pathol. 24, 368 (2020). 33456249 10.4103/jomfp.JOMFP_22_20 PMC7802841

[38] Duddu A. S., Sahoo S., Hati S., Jhunjhunwala S., and Jolly M. K., Multi-stability in cellular differentiation enabled by a network of three mutually repressing master regulators, J. R. Soc. Interface 17, 20200631 (2020). 10.1098/rsif.2020.0631 PMC7536062 32993428

[39] Duddu A. S., Majumdar S. S., Sahoo S., Jhunjhunwala S., and Jolly M. K., Emergent dynamics of a three-node regulatory network explain phenotypic switching and heterogeneity: a case study of Th1/Th2/Th17 cell differentiation, Mol. Biol. Cell 6, ar46 (2022). 10.1091/mbc.E21-10-0521 PMC9265159 35353012

[40] Goodwin B. C., Temporal Organization in Cells: A Dynamic Theory of Cellular Control Processes (Academic Press, London, New York, 1963).

[41] Covert M., Fundamentals of Systems Biology: From Synthetic Circuits to Whole-Cell Models (CRC Press, Taylor & Francis Group, Boca Raton, 2015).

[42] Alon U., An Introduction to Systems Biology: Design Principles of Biological Circuits, 2nd ed. (CRC Press, Taylor & Francis Group, Boca Raton, 2020).

[43] Ferrell J. E., Systems Biology of Cell Signaling: Recurring Themes and Quantitative Models (CRC Press, Boca Raton, FL, 2022).

[44] Hill T. L., Cooperativity Theory in Biochemistry: Steady-State and Equilibrium Systems, 1st ed. (Springer, N.Y., 1977).

[45] Hill T. L., Free Energy Transduction and Biochemical Cycle Kinetics, 1st ed. (Springer, N.Y., 1989).

[46] Ackers G. K., Johnson A. D., and Shea M. A., Quantitative model for gene regulation by lambda phage repressor, Proc. Natl. Acad. Sci. U.S.A. 79, 1129 (1982). 6461856 10.1073/pnas.79.4.1129 PMC345914

[47] Shea M. A. and Ackers G. K., The OR control system of bacteriophage lambda. A physical-chemical model for gene regulation, J. Mol. Biol. 181, 211 (1985). 3157005 10.1016/0022-2836(85)90086-5

[48] Bintu L., Buchler N. E., Garcia H. G., Gerland U., Hwa T., Kondev J., and Phillips R., Transcriptional regulation by the numbers: models, Curr. Opin. Genet. Dev. 15, 116 (2005). 15797194 10.1016/j.gde.2005.02.007 PMC3482385

[49] Bintu L., Buchler N. E., Garcia H. G., Gerland U., Hwa T., Kondev J., Kuhlman T., and Phillips R., Transcriptional regulation by the numbers: applications, Curr. Opin. Genet. Dev. 15, 125 (2005). 15797195 10.1016/j.gde.2005.02.006 PMC3462814

[50] Buchler N. E., Gerland U., and Hwa T., On schemes of combinatorial transcription logic, Proc. Natl. Acad. Sci. U.S.A. 100, 5136 (2003). 12702751 10.1073/pnas.0930314100 PMC404558

[51] Kuhlman T., Zhang Z., Saier M. H. Jr., and T. Hwa, Combinatorial transcriptional control of the lactose operon of *Escherichia coli*, Proc. Natl. Acad. Sci. U.S.A. 104, 6043 (2007). 17376875 10.1073/pnas.0606717104 PMC1851613

[52] Vilar J. M. and Leibler S., DNA looping and physical constraints on transcription regulation, J. Mol. Biol. 331, 981 (2003). 12927535 10.1016/s0022-2836(03)00764-2

[53] Vilar J. M., Guet C. C., and Leibler S., Modeling network dynamics: the *lac* operon, a case study, J. Cell Biol. 161, 471 (2003). 12743100 10.1083/jcb.200301125 PMC2172934

[54] Vilar J. M. G. and Saiz L., Reliable prediction of complex phenotypes from a modular design in free energy space: an extensive exploration of the lac operon, ACS Synth. Biol. 2, 576 (2013). 23654358 10.1021/sb400013w

[55] Hill A. V., The possible effects of the aggregations of the molecules of haemoglobin on its dissociation curves, The Journal of Physiology 40, i (1910).

[56] Hill A. V., The combinations of haemoglobin with oxygen and with carbon monoxide. I, Biochem. J. 7, 471 (1913). 16742267 10.1042/bj0070471 PMC1550542

[57] Yang Z., Rousseau R. J., Mahdavi S. D., Garcia H. G., and Phillips R., The Dynamics of Inducible Genetic Circuits (2025), arXiv:2505.07053 [q-bio.MN]. 10.1103/kzdj-53jf 42141618

[58] Kim J., White K. S., and Winfree E., Construction of an *in vitro* bistable circuit from synthetic transcriptional switches, Mol. Syst. Biol. 2, 68 (2006). 17170763 10.1038/msb4100099 PMC1762086

[59] Martinez-Corral R., Nam K., DePace A. H., and Gunawardena J., The Hill function is the universal Hopfield barrier for sharpness of input-output responses, Proc. Natl. Acad. Sci. U.S.A. 121, e2318329121 (2024). 10.1073/pnas.2318329121 PMC11145184 38787881

[60] Jobe A. and Bourgeois S., *lac* Repressor-operator interaction. VI. The natural inducer of the *lac* operon, J. Mol. Biol. 69, 397 (1972). 4562709 10.1016/0022-2836(72)90253-7

[61] Foster J. B. and Slonczewski J., Microbiology: An Evolving Science, 2nd ed. (W. W. Norton & Co, N.Y., 2010).

[62] Rose J. K. and Yanofsky C., Interaction of the operator of the tryptophan operon with repressor, Proc. Natl. Acad. Sci. U.S.A. 71, 3134 (1972). 10.1073/pnas.71.8.3134 PMC388636 4528605

[63] Razo-Mejia M., Barnes S. L., Belliveau N. M., Chure G., Einav T., Lewis M., and Phillips R., Tuning Transcriptional Regulation through Signaling: A Predictive Theory of Allosteric Induction, Cell Systems 6, 456 (2018). 29574055 10.1016/j.cels.2018.02.004 PMC5991102

[64] Panigrahi A. and O’Malley B. W., Mechanisms of enhancer action: the known and the unknown, Genome Biol. 22, 108 (2021). 33858480 10.1186/s13059-021-02322-1 PMC8051032

[65] Zhang W., Leng F., Wang X., Ramirez R. N., Park J., Benoist C., and Zhang S. H., FOXP3 recognizes microsatellites and bridges DNA through multimerization, Nature 624, 433 (2023). 38030726 10.1038/s41586-023-06793-z PMC10719092

[66] McLure K. G. and Lee P. W., How p53 binds DNA as a tetramer, EMBO J. 17, 3342 (1998). 9628871 10.1093/emboj/17.12.3342 PMC1170672

[67] Ly E., Kugel J. F., and Goodrich J. A., Single molecule studies reveal that p53 tetramers dynamically bind response elements containing one or two half sites, Sci. Rep. 10, 16176 (2020). 32999415 10.1038/s41598-020-73234-6 PMC7528078

[68] Nicolini F., Todorovski T., Puig E., Díaz-Lobo M., Vilaseca M., García J., Andreu D., and Giralt E., How Do Cancer-Related Mutations Affect the Oligomerisation State of the p53 Tetramerisation Domain?, Curr. Issues Mol. Biol. 45, 4985 (2023). 37367066 10.3390/cimb45060317 PMC10296842

[69] Hendrickson W. and Schleif R. F., Regulation of the *Escherichia coli* L-arabinose operon studied by gel electrophoresis DNA binding assay, J. Mol. Biol. 178, 611 (1984). 6387154 10.1016/0022-2836(84)90241-9

[70] Goodrich J. A. and McClure W. R., Regulation of open complex formation at the *Escherichia coli* galactose operon promoters. Simultaneous interaction of RNA polymerase, *gal* repressor and CAP/cAMP, J. Mol. Biol. 224, 15 (1992). 1312605 10.1016/0022-2836(92)90573-3

[71] Yao G., Tan C., West M., Nevins J. R., and You L., Origin of bistability underlying mammalian cell cycle entry, Mol. Syst. Biol. 7, 485 (2011). 21525871 10.1038/msb.2011.19 PMC3101952

[72] Englesberg E., Irr J., Power J., and Lee N., Positive control of enzyme synthesis by gene C in the L-arabinose system, J. Bacteriol. 90, 946 (1965). 5321403 10.1128/jb.90.4.946-957.1965 PMC315760

[73] Lee N., Francklyn C., and Hamilton E. P., Arabinose-induced binding of AraC protein to *araI*_2_ activates the *araBAD* operon promoter, Proc. Natl. Acad. Sci. U.S.A. 84, 8814 (1987). 2962192 10.1073/pnas.84.24.8814 PMC299641

[74] Schleif R., AraC protein, regulation of the L-arabinose operon in *Escherichia coli*, and the light switch mechanism of AraC action, FEMS Microbiol. Rev. 34, 779 (2010). 20491933 10.1111/j.1574-6976.2010.00226.x

[75] Shiau A. K., Barstad D., Loria P. M., Cheng L., Kushner P. J., Agard D. A., and Greene G. L., The structural basis of estrogen receptor/coactivator recognition and the antagonism of this interaction by tamoxifen, Cell 95, 927 (1998). 9875847 10.1016/s0092-8674(00)81717-1

[76] Shang Y., Hu X., DiRenzo J., Lazar M., and Brown M., Cofactor dynamics and sufficiency in estrogen receptor–regulated transcription, Cell 103, 843 (2000). 11136970 10.1016/s0092-8674(00)00188-4

[77] Landman J., Brewster R. C., Weinert F. M., Phillips R., and Kegel W. K., Self-consistent theory of transcriptional control in complex regulatory architectures, PLoS One 12, e0179235 (2017). 10.1371/journal.pone.0179235 PMC5501422 28686609

[78] Elowitz M. and Leibler S., A synthetic oscillatory network for transcriptional regulators, Nature 403, 335 (2000). 10659856 10.1038/35002125

[79] Garcia-Ojalvo J., Elowitz M. B., and Strogatz S. H., Modeling a synthetic multicellular clock: Repressilators coupled by quorum sensing, Proc. Natl. Acad. Sci. U.S.A. 101, 10955 (2004). 15256602 10.1073/pnas.0307095101 PMC503725

[80] Hasty J., Dolnik M., Rottschäfer V., and Collins J. J., Synthetic Gene Network for Entraining and Amplifying Cellular Oscillations, Phys. Rev. Lett. 88, 148101 (2002). 10.1103/PhysRevLett.88.148101 11955179

[81] Mangan S. and Alon U., Structure and function of the feed-forward loop network motif, Proc. Nat. Acad. Sci. U.S.A. 100, 11980 (2003). 10.1073/pnas.2133841100 PMC218699 14530388

[82] Mangan S., Zaslaver A., and Alon U., The coherent feed-forward loop serves as a sign-sensitive delay element in transcription networks, J. Mol. Biol. 334, 197 (2003). 14607112 10.1016/j.jmb.2003.09.049

[83] Mangan S., Itzkovitz S., Zaslaver A., and Alon U., The incoherent feed-forward loop accelerates the response-time of the *gal* system of *Escherichia coli*, J. Mol. Biol. 356, 1073 (2006). 16406067 10.1016/j.jmb.2005.12.003

[84] Carroll S., Grenier J., and Weatherbee S., From DNA to Diversity: Molecular Genetics and the Evolution of Animal Design (Blackwell Science, Malden, MA, 2001).

[85] Santos-Zavaleta A., Salgado H., Gama-Castro S., Sánchez-Pérez M., Gómez-Romero L., Ledezma-Tejeida D., García-Sotelo J. S., Alquicira-Hernández K., Muñiz-Rascado L. J., Peña-Loredo P., Ishida-Gutiérrez C., Velázquez-Ramírez D. A., Del Moral-Chávez V., Bonavides-Martínez C., Méndez-Cruz C. F., Galagan J., and Collado-Vides J., RegulonDB v 10.5: tackling challenges to unify classic and high throughput knowledge of gene regulation in *E. coli* K-12, Nucleic Acids Res. 47, D212 (2019). 30395280 10.1093/nar/gky1077 PMC6324031

